# Brain-to-heart cholinergic synapse-calcium signaling mediates ischemic stroke-induced atrial fibrillation

**DOI:** 10.7150/thno.99065

**Published:** 2024-10-07

**Authors:** Yingran Liang, Gongxin Wang, Siwen Fan, Junyi Zhang, Shuang He, Guixiang Pan, Guoliang Hao, Yan Zhu

**Affiliations:** 1 State Key Laboratory of Component-based Chinese Medicine, Tianjin University of Traditional Chinese Medicine, Beihua South Road, JingHai District, Tianjin 301617, China.; 2 Haihe Laboratory of Modern Chinese Medicine, Tianjin 301617, China.; 3 Henan Academy of Innovations in Medical Science, Institute of Electrophysiology, Zhengzhou 450000, China.; 4 Henan SCOPE Research Institute of Electrophysiology, Kaifeng 475000, China; 5 Second Affiliated Hospital of Tianjin University of Traditional Chinese Medicine, Tianjin 300250, China.

**Keywords:** atrial fibrillation detected after stroke, ischemic stroke-induced AF, cholinergic synapse signaling, Ca^2+^ signaling, Stroke heart syndrome, ischemic stroke, Wenxin Keli

## Abstract

**Background:** Stroke-related cardiovascular diseases have attracted considerable attention, with atrial fibrillation (AF) being among the most frequent complications. Despite increasing clinical evidence, experimental models of stroke-induced AF are still lacking, hindering mechanistic discoveries and the development of adequate therapeutics targeting this stroke-heart syndrome (SHS). This study aims to create a rat model of ischemic stroke-induced AF (ISIAF) and to explore the efficacy and mechanism of Wenxin Keli (WK), an antiarrhythmic Chinese medicine.

**Method:** The middle cerebral artery occlusion/reperfusion model was adapted to create subacute brain ischemia in rats with normal cardiac function. Invasive electrophysiologic studies and *ex vivo* optical mapping were performed to evaluate the altered electrophysiological parameters and Ca^2+^ handling properties. RNA-seq analysis, RT-PCR, and immunohistochemistry (IHC) with immunofluorescence (IF) were employed to assess the SHS model and elucidate the mechanisms of ISIAF and the effects of WK. UPLC/Q-TOF-MS, molecular docking, and whole-cell patch recordings were used to identify the active components of WK for SHS.

**Results:** Ischemic stroke aggravated atrial electrical instability, altered action potential duration (APD), Ca^2+^ transient duration (CaT), conduction heterogeneity, and spatially discordant alternans in SHS rat hearts. These abnormalities were alleviated by WK. RNA-seq analysis revealed that M_3_-mediated cholinergic synapse signaling and L-type calcium channel (LTCCs)-mediated Ca^2+^ signaling play prominent roles in ISIAF development and its reversal by WK. UPLC/Q-TOF-MS analysis identified 19 WK components as the main components in plasma after WK treatment. Molecular docking screening identified Dioscin as the major active component of WK. WK and Dioscin reduced I_Ca-L_ in a concentration-dependent manner with a half-maximal inhibitory concentration of 24.254 ± 2.051 mg/mL and 8.666 ± 0.777 µmol/L, respectively.

**Conclusion:** This study established an experimental model of ISIAF capable of characterizing clinically relevant atrial electrophysiological changes post-cerebral ischemia. Molecular mechanistic studies revealed that the cholinergic-calcium signaling pathway is central to this brain-heart syndrome. Ischemic stroke-induced atrial fibrillation is partially reversible by the Chinese medicine Wenxin Keli, which acts via regulation of the cholinergic-calcium signaling pathway, with its active component Dioscin directly binding to I_KM3_ and inhibiting I_Ca-L_.

## Introduction

Physiological and pathological connections between the nervous and cardiovascular systems are well established 1-4. More than 1.5 million deaths worldwide are attributed to neurocardiogenic mechanisms, including post-stroke cardiovascular complications 5,6. Clinically referred to as "stroke heart syndrome" (SHS) 7, post-stroke cardiac events are linked to pathophysiological processes known as "stroke-induced heart injury" (SIHI) 8,9. Specifically, atrial fibrillation (AF) is more common in acute ischemic stroke (AIS) patients, occurring at a rate 8 times higher than that of similarly matched individuals without a history of stroke 10,11. Meta-analysis indicates that progressively integrating cardiac monitoring during long-term follow-up can detect previously undetected AF in about 25% of stroke patients 12,13. AF detected after stroke (AFDAS) may arise from primary cardiogenic, secondary neurogenic, or mixed causes 1,14. The interaction of underlying cardiogenic processes and stroke-related neurogenic events theoretically leads to AFDAS. Since these pathophysiological processes can both generate and perpetuate AFDAS through AF-related atrial remodeling, it is crucial to understand the underlying mechanisms in appropriate experimental models 15,16.

A dysfunctional autonomic nervous system (ANS) plays a critical role in the pathophysiology of AF 17. The intrinsic cardiac nervous system (ICNS) comprises ganglionated plexi (GPs), interconnected ganglia, and axons. These ganglia contain various neuronal components, including adrenergic, cholinergic, afferent, and linking neurons 18,19. All neurons in the parasympathetic nervous system produce and utilize acetylcholine (ACh) for neurotransmission 20,21. The cardiomotor function of this cholinergic system helps slow the heart rate, reduce blood pressure, and balance the overexcitation of the sympathetic nervous system. Within and between neuronal networks and the heart, ACh initiates neurotransmission 18,22. It is secreted by stimulated postganglionic nerves and binds to muscarinic ACh receptors (mAChR) on cardiac cell plasma membranes 23. However, the delayed rectifying K^+^ current is activated by the M3 muscarinic acetylcholine receptors trigger delayed rectifying K^+^ currents (I_KM3_), which has pro-dysrhythmic (facilitating AF) and negative chronotropic (participating in cardiac repolarization) effects 24,25. In addition, the ACh-activated potassium current (I_K-ACh_) is present only in the atria of humans and most animals, making it a promising target for atrium-specific medication 24.

Intracellular Ca^2+^ homeostasis also plays a key role in the maintenance of AF 26,27. Altered Ca^2+^ handling affects sensitive ion channels, as shown in recent studies, including ryanodine receptors 2 (RyR_2_) located at the sarcoplasmic reticulum Ca^2+^-ATPase (SERCA) 28. The trigger is the mishandling of Ca^2+^ by the sarcoplasmic reticulum (SR), initiating Ca^2+^ waves, eliciting Ca^2+^ transients, and causing delayed afterdepolarizations (DADs) 29. Additionally, the enhanced calcium content in the SR, due to Ca²⁺ mishandling, enables an excessive response to additional stimuli, such as burst pacing or triggered impulses 30. On the substrate level, Ca^2+^ mishandling promotes the inactivation of Ca_V1.2_ L-type calcium channels (LTCCs). However, Ca^2+^ influx via LTCCs in the heart serves as a multipurpose signal that affects action potential duration (APD), activates muscle contraction, and modulates gene expression. The heart's use of LTCC Ca^2+^ as a multidimensional signaling molecule is made more complex by various physiological factors 31,32.

Traditional Chinese medicine (TCM) has been a rich source for discovering and developing multitarget cardiovascular medicine 33. Wenxin Keli (WK) is the first antiarrhythmic Chinese medicine approved by the China Food and Drug Administration. It is composed of Codonopsis Radix (Dang Shen), Polygonati Rhizoma (Huang Jing), Notoginseng Radix et Rhizoma (San Qi), Ambrum (Hu Po), and Nardostachyos Radix et Rhizoma (Gan Song). WK has been widely used for managing arrhythmias in China. Reports suggest that WK is effective in treating cardiac arrhythmia through complex multichannel inhibition and regulation of transient potassium outward current (I_to_), late sodium current (I_Na-L_), and L-type calcium current (I_Ca-L_) 34-36. However, whether WK is effective for AFDAS and its mechanism of action remains unclear.

Therefore, to study AFDAS, we created a rat model of ischemic stroke-induced AF (ISIAF), characterized its electrophysiological properties, and evaluated the therapeutic efficacy of WK using optical mapping and whole-cell patch clamp techniques. We also identified underlying mechanisms using RNA-seq and network pharmacology analyses. Our study shows that ischemic stroke-induced cardiac dysfunction includes AF, and this pathological process develops at least in part via a previously undocumented cholinergic synapse and calcium signaling mechanism in the atrium. WK exerts anti-cardiac remodeling effects by inhibiting the cholinergic signaling and calcium signaling pathways, alleviating ISIAF.

## Materials and methods

### Drugs and reagents

WK was obtained from Shandong Buchang Pharmaceutical Co., Ltd (Shandong, China). Metoprolol (Met) was purchased from AstraZeneca Pharmaceutical Co., Ltd (London, United Kingdom). Ginsenoside Rb1 (41753-43-9), Ginsenoside Re (52286-59-6), Ginsenoside Rg1 (22427-39-0), Atractylenolide II (73069-14-4), Tanshinone-IIA (568-72-9), Cryptotanshinone (35825-57-1), Nardosinone (23720-80-1) and Dioscin (19057-60-4) were purchased from Chengdu Lemeitian Pharmaceutical Technology Co., Ltd (Chengdu, China). Blebbistatin (ab120425) and Rhod-2AM (ab142780) were obtained from Abcam Plc (Cambridge, United Kingdom). F127 (P3000MP) was purchased from Invitrogen (Carlsbad, United States). Rh237 (Sc-499456) was obtained from Santa Cruz Biotechnology (Beijing, China). Nifedipine (21829-25-4, Nif) was obtained from Sigma-Aldrich (Missouri, United States). Bestar™ SybrGreen qPCR MasterMix was purchased from Shanghai Kolaman Reagent Co., Ltd (DBI- 2043, Shanghai, China). Transcriptor First Strand cDNA Synthesis Kit was obtained from Roche (04897030001, Mannheim, Germany). Anti-CHRM3 (bs-1289R) antibodies were purchased from Bioss (Beijing, China).

### Animal and drug treatment

All animal experimentation protocols were developed in accordance with the principles outlined in the Basel Declaration and the guidelines for the Care and Use of Laboratory Animals established by the Ministry of Science and Technology of China. These protocols were executed following approval from the Laboratory Animal Ethics Committee of the Henan SCOPE Research Institute of Electrophysiology (License number: SGLL20221031142). Male Sprague-Dawley (SD) rats (240-260 g) were purchased from Beijing Sipeifu Biotechnology Co., Ltd. (Beijing, China) and were kept in well-ventilated polypropylene cages with a regulated internal temperature of 22 ± 2°C and humidity of 40 ± 5% and maintained in a 12 h light/dark cycle. Clean water and a commercial regular rat diet (Beijing Sipeifu Biotechnology Co., Ltd.) were offered without restriction. Animals were fasted for 12 h before surgery while still given unrestricted access to water. The behavioral evaluation was carried out when the rats were most active.

SD rats were randomly divided into six groups, including Sham, MCAO (Model), Met (54 mg/Kg, positive control, approximately 6 times the human clinical dose) + MCAO, WK low dose (1.35 g/Kg, approximately the human clinical dose) + MCAO, middle dose (4.05 g/Kg, approximately 3 times the human clinical dose) + MCAO, and high dose (8.1 g/Kg, approximately 6 times the human clinical dose) + MCAO groups. WK and Met were administered orally in their respective dose once daily for 14 days 37.

### Middle Cerebral Artery Occlusion (MCAO) model

Rats were given 4% isoflurane mixed with 70% nitrous oxide and 30% oxygen before surgery. A small animal anesthetic machine (Matrix VIP 3,000; Midmark, United States) was used to maintain anesthesia while lowering the isoflurane concentration to 2.5%. The rats were maintained at 37℃ on a heating blanket throughout the surgical operation. A widely recognized middle cerebral artery occlusion surgery was performed to establish the cerebral ischemia model, as we described previously 38. In brief, after an orderly exposure of the left common carotid artery (CCA), external cerebral artery (ECA), and internal cerebral artery (ICA), a nylon monofilament (Reward Co., Ltd., Guangzhou, China) was gently guided towards the ICA by inserting it into the left ECA. After 60 minutes of MCAO, nylon monofilament was removed to restore blood flow perfusion.

### Langendorff perfused rat hearts and electrophysiological mapping

After 15 minutes of isoflurane anesthesia, the SD rat was intraperitoneally injected with heparin (3125 U/kg) and subsequently euthanized 15 to 20 minutes later. Immediately following euthanasia, the chest was opened, and the heart was flushed with cold K-H buffer and heparin and instantly transferred into a prepared Langendorff perfusion apparatus. The K-H buffer contained (in mM) 119 NaCl, 4 KCl, 1.8 CaCl_2_, 1 MgCl_2_, 1.2 NaH_2_PO_4_, 25 NaHCO_3_, and 10 glucose (pH 7.4). To get the heart back to its regular rhythm, all the remaining blood was evacuated. All experiments were performed after 15 min of heart standardization. Stimulation electrodes were inserted in the atria. Mapping Lab matrix multichannel electrodes were in contact with the atria epicardium, and ECG electrodes were placed in the right atrium and left ventricle of the heart. ECG II lead signal, string stimulation, S1S2 stimulation, and S1S1 stimulation signals were detected.

### Optical mapping for *ex vivo* electrophysiology

The rat heart was rapidly transferred to a prepared Langendorff perfusion apparatus (37°C at 10 mL/min). To prevent motion artifacts during optical recording, Blebbistatin (10 µmol/L), a compound that uncouples excitation and contraction, was included in the perfusate 39 to prevent motion artifacts during optical recording. Rhod-2AM (100 mL of 1 mg/L in DMSO containing 20% pluronic acid) was applied for 30 min, and RH237 (10 mL of 5 mg/mL in DMSO) was used for 5 min. Two LED light sources operating at 530 ± 25 nm and bandpass filtered from 511 to 551 nm (LEDC-2001, MappingLab, United Kingdom) provided the excitation light. An objective was used to gather the right atria's emission light, and a dichroic mirror was used to divide the signals at 630 nm. Signals from cardiac Ca^2+^ were filtered at 590 nm, whereas Vm signals were filtered at 700 nm. The imaging systems used were two CMOS cameras (OMS-PCI-2002, MappingLab, United Kingdom) with a 0.9 kHz sampling rate enabled to obtain fluorescence signals from the heart 40.

Data analysis was conducted using OMapScope5.0 software (MappingLab, United Kingdom). Optical signals were spatially aligned and processed using 3×3-pixel Gaussian spatial filters. The maximal departure speed of the APD and Ca^2+^ upstroke was used to calculate the activation time. APD90 was calculated as the time from maximal upstroke velocity (dF/dt_max_) to 90% repolarization. To assess the atrial effective refractory period (AERP), an extra-stimulus (S1S2; 30 S1 stimuli at 6Hz followed by a premature S2 stimulus ranging from 150-30 ms) protocol was implemented. To induce APD and Ca^2+^ transient duration (CaT) alternans, continuous pacing at progressively faster frequencies between 100 ms to 20ms (S1S1) was performed. Short-long APD sequences (or low-high CaT sequences) were classified as negative during the alternans phase, whereas long-short APD sequences (or high-low CaT sequences) were classified as positive 41. APD/CaT alternans were discovered using the spectral approach, which is consistent with previously described programs 42. The ratio of the average smaller beat release amplitude to the average bigger beat release amplitude was used to compute the magnitude of CaT alternans, which is equal to 1 minus that number. The Ca^2+^ transient refractoriness was evaluated using the S1S2 protocol to quantify CaT recovery from refractoriness. At S1S2 coupling intervals ranging from 150 to 30 ms for each condition, the S2/S1 CaT ratio, which is the ratio of the S2-induced CaT amplitude to the S1-induced CaT amplitude, was calculated. At various S1S2 coupling intervals for each condition, maps of the S2/S1 CaT ratio were created. Plotting the S2/S1 CaT ratio against the relevant S1S2 coupling period allowed for the construction of the Ca^2+^ release restitution curves. In four locations, Ca^2+^ release restitution curves were constructed 40,43.

### RNA sequencing and Ingenuity^Ⓡ^ pathway analysis (IPA)

Total RNA was extracted from the atria tissue, and its purity and quality were examined. The NEBNextⓇ UltraTM RNA Library Prep Kit (New England Biolabs, Ipswich, MA, United States) was used to create the library for transcriptome sequencing on the Agilent 2100 bioanalyzer. The built-in library was then sequenced using the Illumina Novaseq platform. HISAT2 alignment software (John Hopkins University, Baltimore, Maryland, United States) was used to quantify the sequencing data. Differential expression analyses among the Sham, Model, and WK groups were carried out using the DESeq2 R tool. The findings were subjected to multiple testing corrections employing the False Discovery Rate approach, ensuring that the threshold for adjusted P-value significance was maintained at or below 0.05. The intersection genes from the Model vs. Sham and WK vs. Model groups were identified and selected based on a |fold change| ≥ 1.25 (log fold changes calculated using Log2) and an adjusted P-value ≤ 0.05. The gene IDs, fold changes, and adjusted P-values were then input into the Gene Ontology (GO) and Kyoto Encyclopedia of Genes and Genomes (KEGG) databases within the Ingenuity Pathway Analysis (IPA) software for core analysis.

### Molecular docking

The PDB database (http://www.rcsb.org/) and UniProt database (https://www.uniprot.org/) were used to obtain three-dimensional structures of the gene of interest that serve as protein receptors. Two-dimensional structures of the main WK chemical compounds (identified as ligands by UPLC/Q-TOF-MS) were downloaded from the PubChem database and converted into three-dimensional structures using Chem3D software. Hydrogenation of proteins using AutoDock Tools1.5.6, hydrogenation of small molecules, and determination of torsional bonds were performed and saved as a pdbqt file. The molecular docking range parameters were set using a Grid plate, the docking mode was set as semi-flexible docking, and the docking algorithm was set as the Lamarck genetic algorithm. Molecular docking free energies and docking results data files were saved after running the Auto Dock Vina1.2.0 application.

### Quantitative real-time polymerase chain reaction (qRT-PCR)

Total RNA was isolated from cardiac tissue using the TRIzolTM reagent. The cDNA was synthesized by reverse transcription using the Transcriptor First Strand cDNA Synthesis Kit. The PCR reaction was performed with Bestar^TM^ SybrGreen qPCR MasterMix. Primers for the genes to be detected include CHRM1, INA, SLC6A15, SYT4, CHAT, SLC5A7, CHRM4, CACNA1B, ACHE, CACNB1, ATP2A3, GNG3 and CHRM3 (sequences listed in supplementary data table 1), which were synthesized by Sangon Company (Shanghai, China).

### Cell viability assay and Immunocytofluorescence (IF)

Mouse HL-1 cardiomyocytes were grown in a 10-cm petri dish with 1% penicillin-streptomycin (PS) and 10% fetal bovine serum in Dulbecco's modified eagle medium, which was maintained at 37°C and 95% air/5% CO_2_. A 96-well plate containing HL-1 was seeded with 104 cells per well and cultivated for 24 h in complete media with various doses of Dioscin (Dio, 0.2, 1, 5, 20, 100, 200, and 500 μmol/L). Each well received 10 µL of CCK-8 solution, which was then incubated at 37°C for 4 h to measure cell viability using a microplate reader. For IF detection, HL-1 cells were placed on a 96-well plate, fixed with 4% paraformaldehyde solution, blocked with 5% FBS for 2 h, incubated with the primary antibody (CHRM3, 1:200) overnight at 4°C, washed with PBS-T, and then incubated for 2 h at room temperature with the corresponding fluorescent secondary antibody and Hoechst 33342. Following wash with PBS-T, HL-1 cell numbers, and fluorescence intensities were determined with the Operetta high-content analysis system (PerkinElmer Inc., USA).

### Cell isolation and electrical recordings

Male rat atrial myocytes were enzymatically separated using previously established techniques 44. The rat heart was rapidly removed and placed in Ca^2+^-free Tyrode solution (in mM: 135 NaCl, 1.0 MgCl_2_, 5.4 KCl, 0.33 NaH_2_PO_4_, 10 HEPES, and 10 glucose, pH 7.4) at 0°C. Retrograde aortic perfusion and aortic intubation were carried out by Ca^2+^-free Tyrode solution (8 mL/min) into the heart for 5 min, followed by 20 to 25 min treatment at 37 °C with an enzymatic solution containing collagenase type II (0.46 mg/mL) and bovine serum albumin (1 mg/mL). Following perfusion, several small pieces of the heart were put in a beaker filled with KB solution (in mM: 70 KOH, 20 KH_2_PO_4_, 40 KCl, 20 taurine, 50 glutamic acid, 0.5 EGTA, 10 HEPES, 10 glucose, and 3.0 MgSO4, pH 7.4). Filtration was used to get rid of the undigested tissue from the atrial myocytes. Before measurement, KB solution (bubbled with 100% O_2_) filled with cells was maintained at room temperature (23-25 °C) for at least 1 h. The Ca^2+^ current was recorded using a whole-cell patch clamp 45. Pipettes were pulled in a pipette puller (Sutter Instruments, Novato, CA, USA) containing intracellular solution with a resistance of 2-5 MΩ. The pipette was filled with the intracellular pipette solution. The whole-cell allocation was achieved through gigabit and membrane disruption. Membrane capacitance was estimated from the capacitive transient. The pClamp 10.0 software and an axon patch 200 B amplifier (Axon Instruments, Union City, CA, USA) were used to record data. The extracellular solution contained (in mM) 135 NaCl, 5.4 CsCl, 1.8 CaCl_2_, 1 MgCl_2_, 0.3 BaCl_2_, 0.33 NaH_2_PO_4_, 10 glucose and 10 HEPES (pH 7.4). The electrode solution contained (in mM) 120 CsCl, 5 Na_2_ATP, 5 MgCl_2_, 1 CaCl_2_, 10 TEA-Cl, 10 EGTA and 10 HEPES (pH 7.4). The Na^+^ channel was inactive from holding potential -80 mV of first polarized to 40 mV for 50 ms.

### Measurement of intracellular calcium

The cardiomyocytes were equilibrated at room temperature for 1 h and then introduced into a calcium imaging solution (1.8 mM Ca^2+^, three times, 20-30 min each). Following calcium loading, the calcium indicator 2 μM Rhod-2 AM and Pluronic F127 (0.02%) were directly added to the myocyte suspension and incubated for 30 min at room temperature to allow complete de-esterification. Excess dye was removed by washing once with calcium imaging solution. The myocyte suspension was subsequently placed at 4°C to reduce oxygen consumption and placed in a chamber connected to an inverted microscope. At room temperature, the calcium imaging solution was continuously perfused with a circulating bench-top solution containing calcium (1.8 mM Ca^2+^) at a rate of 2 mL/min in 95% O_2_ / 5% CO_2_.

Calcium signals were detected approximately 5 minutes after the cardiac myocytes had settled. Immediately measure the control cardiac myocytes, and then measure the efficacy of WK on cardiac myocytes incubated with WK 20 mg/mL for 2 min. When searching for cells, electrical stimulation (10-20 V) is usually applied first to select cells that contract in correspondence with the stimulation frequency. Once the signal is officially recorded, to reduce fluorescence quenching and phototoxicity, calcium signals are typically activated with light for 5-6 seconds at a time, with red light simultaneously turned on for electrical stimulation. A camera captures the calcium transient signals.

To assess the function of RyR_2_ and SERCA, Rhod-2-loaded cardiomyocytes were exposed to a Tyrode solution containing different components. In 0 Ca^2+^ and 0 Na^+^ solutions containing caffeine, RyR_2_ function was assessed by caffeine-induced Ca^2+^ transients. SERCA recovery was evaluated in 0 Ca^2+^ and 0 Na^+^ solutions containing tetracaine (RyR_2_ blocker). Moreover, the function of RyR_2_ and SERCA is fitted by a polynomial, and the rate constant is obtained 46-48.

### Liquid chromatography of WK ingredients in rat plasma

Blank rat blood samples were collected via ocular venous plexus. Six rats were given an oral dosage of WK aqueous solution (10 times the therapeutic dose) and were given unrestricted access to water during the blood collection process. Blood samples were taken at 15-, 30-, and 60-min following WK dosing. Plasma was extracted from the blood samples by centrifugation at 6000 rpm for 10 min after they were placed into heparinized polypropylene tubes. Plasma samples were kept at -80°C till use. In brief, 1 mL of acetonitrile was added to 250 μL of rat plasma, vortexed for 3 min, and centrifuged for 10 min at 13,000 rpm. The resulting supernatant was then moved into a sterile tube and dried at 37°C using nitrogen gas. After the residue was reconstituted in 100 μL of methanol, it was vortexed for 3 min and centrifuged for 10 min at 13,000 rpm. The UPLC/Q-TOF-MS apparatus was filled with the supernatant. A COTERST T3 column (2.1×150 mm, 1.6 m) was used for chromatographic separation, with a column temperature of 40°C and a 2 μL injection volume. The mobile phase consists of solvent A (0.1% formic acid deionized water) and solvent B (acetonitrile). Samples were eluted with a flow rate of 0.3 ml/min using a gradient elution program with the following times: 0-5 min (95-80% A); 5-30 min (80-50%A); 30-35 min (50-20%A); 35-38 min (20-20%A); and 38-40 min (20-95% A). To achieve MS detection, a high-definition MS system (Waters, United States) with positive and negative electrospray modes was utilized. The WK components were preliminarily identified by matching retention time (RT), m/z values, and MS/MS fragments within the database resource. The precise molecular information and secondary fragment information of WK chemical composition were obtained by Q/TOF-MS detection, and then the composition identification was achieved by a one-by-one comparison with the chemical composition database.

### Statistical analysis

Data are presented as mean ± SD. When necessary, the Mann-Whitney U test or an unpaired Student's t-test was used to compare the two groups. Tukey's or Bonferroni's multiple comparisons test was used to compare the results of experiments involving more than two groups. Data analysis tools included OriginPro 2018 (OriginLab Corporation, Northampton, MA, United States), GraphPad Prism 7 (GraphPad Software Inc., San Diego, California, United States), and Clampfit 7.0 (Axon). Statistics were considered significant for P-values under 0.05.

## Results

### Ischemic stroke caused sinus arrhythmia that was suppressed by WK

**Figure 1:**
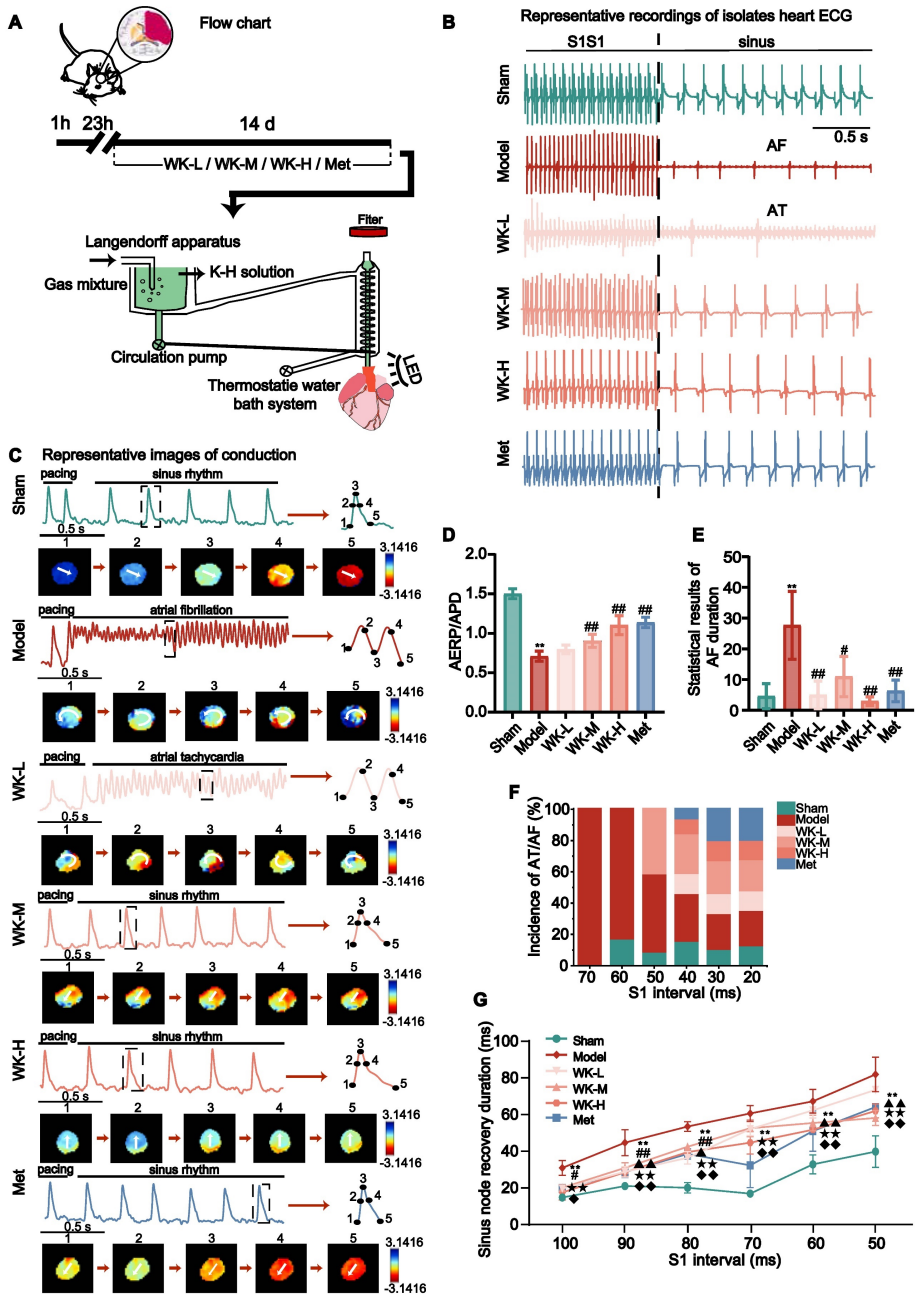
**Ischemic stroke caused sinus arrhythmia that was suppressed by WK.** (A) Timeline of the MCAO operation, drug treatment, and the following *ex vivo* cardiac optical mapping. (B) Representative ECG recordings of isolated heart during sinus rhythm. (C) Representative images of conduction in Sham, Model, WK-L, WK-M, WK-H and Met groups under the burst pacing at S1S1. (D) AERP/APD of each group. (E) Statistical results of AF duration in each group. Values were expressed as mean ± SD (n=3-5, ^*^P < 0.05 and ^**^P < 0.01, Model vs. Sham; ^#^P < 0.05 and ^##^P < 0.01, Model vs. WK) (F) Incidence of atrial ectopy or fibrillation for each S1 interval. (G) Sinus node recovery duration of each group under different stimulation frequencies (100-50 ms). Values were expressed as mean ± SD (n=3-5, ^*^P < 0.05 and ^**^P < 0.01, Model vs. Sham; ^#^P < 0.05 and ^##^P < 0.01, Model vs. WK-L; ^▲^P < 0.05 and ^▲▲^P < 0.01, Model vs. WK-M; ^★^P < 0.05 and ^★★^P < 0.01, Model vs. WK-H; ^◆^P < 0.05 and ^◆◆^P < 0.01, Model vs. Met).

The cerebral infarction was assessed in 2,3,5-Triphenyltetrazolium chloride (TTC)-stained brain slices. Following MCAO surgery, infarct volume in the Model increased by 26.21 ± 2.38% (p < 0.01) compared to that of the Sham (**Supplemental Figure 1**). As shown in the experimental scheme in **Figure 1A**, after 60 min ischemia, rats were treated with different doses of WK or a positive control drug Met for 14 days. ECG from Sham, Model, WK-L, WK-M, WK-H, and Met-treated groups (**Figure 1B**) indicated that cerebral ischemia caused clear arrhythmia, which was suppressed by Met or WK in a dose-dependent manner. Optical AP traces shown in **Figure 1C** further demonstrated sinus rhythm and circular reentry abnormality after ischemic stroke, while Met or WK improved it in a dose-dependent manner. Similarly, S1S1 activation caused Model rat hearts to exhibit more frequent atrial tachycardia (AT)/AF (Sham = 4.60 ± 4.10 vs. Model = 27.67 ± 11.02, P < 0.01, **Figure 1E**) and decrease the atrial effective refractive period (AERP)/APD (Sham = 1.50 ± 0.06311 vs. Model = 0.71 ± 0.06, P < 0.01, **Figure 1D**), which was reduced after treatment with WK-L, WK-M, WK-H, and Met to 0.80 ± 0.05 (P > 0.05), 0.90 ± 0.08 (P < 0.01), 1.10 ± 0.12 (P < 0.01) and 1.14 ± 0.07 (P < 0.01), respectively. In contrast to Sham rats, Model rats had a startlingly longer overall AT/AF duration (P < 0.01 or P < 0.05) and a higher likelihood of inducing AT/AF (P < 0.01 or P < 0.05). In addition, Model rats also improved their susceptibility to AF. The percentage of Model rats that had AF started to appear at 70 ms, and when the frequency increased, the incidence of AF in the Model rats persisted. However, WK-L, WK-M, WK-H and Met groups exerted an anti-arrhythmic function by reducing AT/AF duration (WK-L = 5.00 ± 4.59, P < 0.01; WK-M = 11.00 ± 6.56, P < 0.05; WK-H = 3.00 ± 1.00, P < 0.01 and Met = 6.33 ± 3.51, P < 0.01), and increasing the AERP/APD susceptibility (P < 0.01 or P < 0.05, **Figures 1D-F**).

In S1S1 pacing, sinus node recovery duration was longer, and sinus arrhythmia was more likely to occur, resulting in an increased incidence of AF/AT in the Model rats. Compared with Sham rats, the sinus node recovery duration was prolonged at 100-50ms stimulation (Sham = 11.90 ± 1.76 vs. Model = 26.50 ± 3.69, P < 0.01; Sham = 17.50 ± 1.25 vs. Model = 39.00 ± 6.46, P < 0.01; Sham = 16.67 ± 2.60 vs. Model = 47.00 ± 2.44, P < 0.01; Sham = 13.75 ± 1.25 vs. Model = 53.55 ± 3.88, P < 0.01; Sham = 28.25 ± 4.73 vs. Model = 59.55 ± 5.84, P < 0.01; and Sham = 34.60 ± 7.87 vs. Model = 72.85 ± 8.64, P < 0.01, respectively at 100-50ms, down stepped 10ms each), reduced in the WK and Met-treat rats (WK-L = 16.25 ± 1.25, P < 0.05; 24.58 ± 2.60, P < 0.01; 31.56 ± 3.13, P < 0.01; 45.75 ± 3.32, P > 0.05; 55.00 ± 3.75, P > 0.05; and 65.17 ± 1.01, P > 0.05; WK-M = 16.67 ± 1.44, P > 0.05; 26.56 ± 2.58, P < 0.01; 37.00 ± 0.74, P < 0.05; 46.17 ± 4.30, P > 0.05; 48.75 ± 3.85, P < 0.01; and 51.25 ± 3.68, P < 0.01; WK-H = 14.38 ± 3.75, P < 0.01; 24.38 ± 2.17, P < 0.01; 34.38 ± 0.72, P < 0.01; 39.06 ± 5.63, P < 0.01; 45.42 ± 1.44, P < 0.01; and 54.75 ± 3.47, P < 0.01; Met = 16.25 ± 2.04, P < 0.05; 24.38 ± 2.65, P < 0.01; 33.42 ± 2.50, P < 0.01; 27.75 ± 11.05, P < 0.01; 44.75 ± 10.04, P < 0.01; and 56.67 ± 0.72, P < 0.01, respectively at 100-50ms, down stepped at 10ms each **Figure 1G**).

### Ischemic stroke-induced APD alterations that were reversed by WK

Under sinus rhythm, cerebral ischemia caused significant changes in a series of AP indicators. APD90 in the Model was longer than that in the Sham (Sham = 36.53 ± 2.11 vs. Model = 85.42 ± 7.66, P < 0.01), while APD90 repolarization in the WK or Met-treatment rats was reduced compared to that in the Model (WK-L = 57.58 ± 2.39, P < 0.01; WK-M = 56.70 ± 5.06, P < 0.01; WK-H = 55.88 ± 7.72, P < 0.01; and Met = 61.32 ± 4.62, P < 0.01, **supplemental Figure 2A**). Compared to the Sham, the rise time (Sham = 12.51 ± 0.39 vs. Model = 26.21 ± 1.20, P < 0.01) and velocity (calculated as previously described 40,49, Sham = 7.97 ± 1.05 vs. Model = 2.92 ± 0.63, P < 0.01) following an ischemic stroke were considerably delayed in the event of sinus rhythm. When compared to the Model, the WK or Met-treatment showed some improvement (WK-L = 16.29 ± 0.67, P < 0.01, and 3.83 ± 0.86, P > 0.05; WK-M = 16.47 ± 0.77, P < 0.01, and 4.49 ± 0.32, P < 0.05; WK-H = 14.90 ± 1.04, P < 0.01, and 5.57 ± 0.64, P < 0.01; Met = 17.09 ± 1.02, P < 0.01, and 5.01 ± 0.79, P < 0.01, respectively **supplemental Figure 2B-C**). APD90 interquartile interval (IQR) in the Model differed substantially from that of the Sham (Sham = 4.73 ± 0.95 vs Model = 21.04 ± 0.62, P < 0.01), while the WK or Met-treatment relieved the discrepancy (WK-L = 13.67 ± 2.70, P < 0.01; WK-M = 12.07 ± 0.93, P < 0.01; WK-H = 9.59 ± 1.91, P < 0.01; and Met = 12.04 ± 2.61, P < 0.01, **supplemental Figure 2D**). Moreover, APD repolarization to 30% / APD repolarization to 80% (APD30/80, Sham = 0.49 ± 0.01 vs Model = 0.35 ± 0.02, P < 0.01) ratios significantly changed as a result of cerebral ischemia, which was improved by WK or Met-treatment (WK-L = 0.39 ± 0.01, P > 0.05; WK-M = 0.39 ± 0.01, P > 0.05; WK-H = 0.40 ± 0.04, P < 0.01; Met = 0.40 ± 0.01, P < 0.05, **Supplemental Figure 2E**).

**Figure 2:**
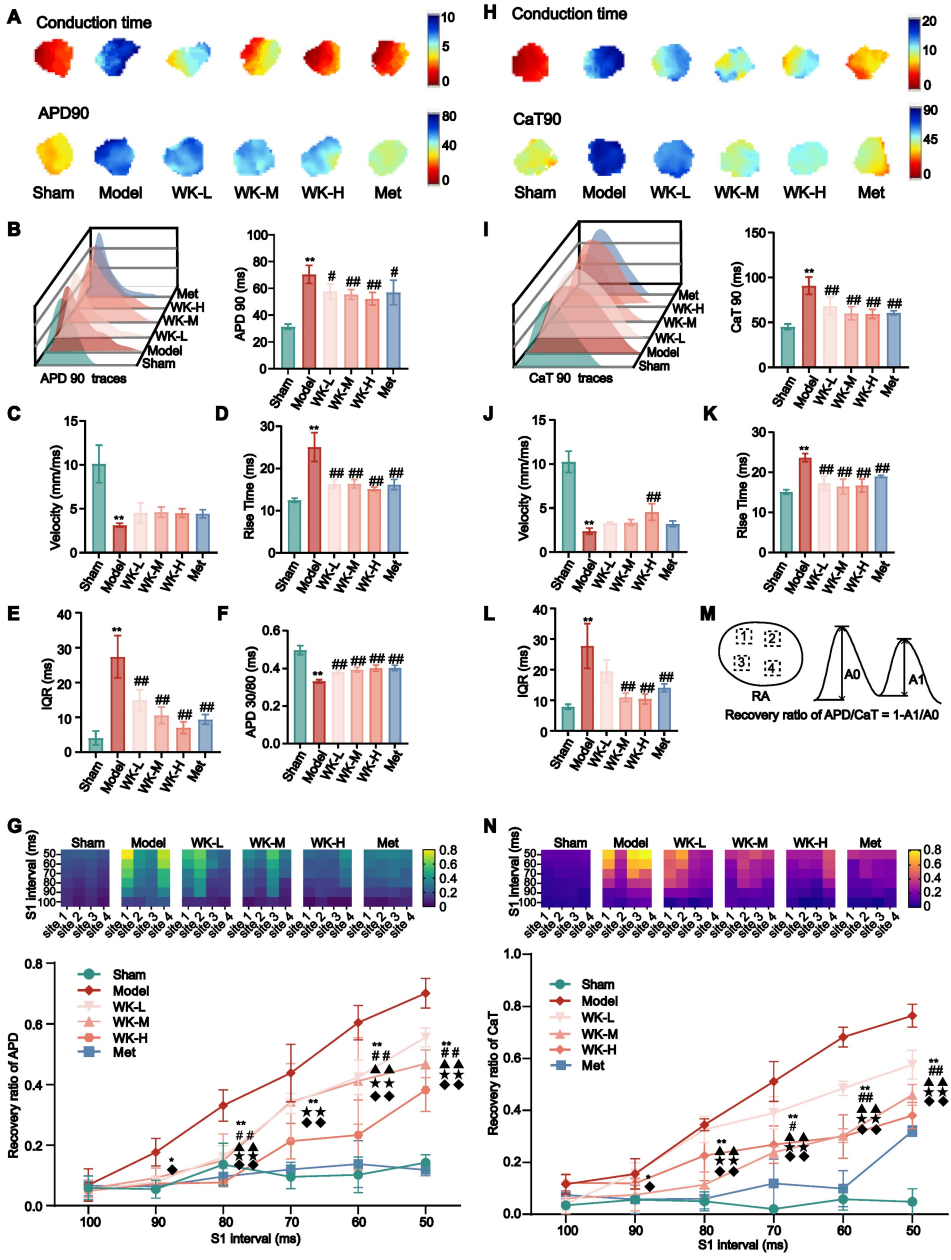
** APD and CaT alterations in ISIAF before and after WK treatment via *ex vivo* optical mapping.** (A) Conduction and APD90 maps with four recording sites of six groups under 6Hz. (H) Conduction and CaT90 maps with four recording sites of six groups under 6Hz. (B, I) APD90 and CaT90 traces and quantification in each group. (C, J) Calculation of velocity in AP phase and CaT phase in each right atrium. (D, K) Calculation of rise time in AP phase and CaT phase in each right atrium. (E, L) Calculation of IQR in AP phase and CaT phase in each right atrium. (F) Statistical results of APD 30/80. (M) Calculation diagram of APD/CaT alternans. Values were expressed as mean ± SD (n = 3-5). ^*^P < 0.05 and ^**^P < 0.01, Model vs. Sham;^ #^P < 0.05 and ^##^P < 0.01, Model group vs. WK groups. (G, N) The heat maps showed the different conduction rates of the six groups in APD and CaT. Depiction of recovery ratio of APD and CaT at the integral level. Values were expressed as mean ± SD (n=3-5, ^*^P < 0.05 and ^**^P < 0.01, Model vs. Sham; ^#^P < 0.05 and ^##^P < 0.01, Model vs. WK-L; ^▲^P < 0.05 and ^▲▲^P < 0.01, Model vs. WK-M; ^★^P < 0.05 and ^★★^P < 0.01, Model vs. WK-H; ^◆^P < 0.05 and ^◆◆^P < 0.01, Model vs. Met).

A 6Hz stimulation was used to correct the conduction differences due to differences in sinus rhythm. Atrial conduction maps of AP and atrial APD90 maps (**Figure 2A-B**) indicated that cerebral ischemia caused clear APD90 extension, which was suppressed by Met or WK. Specifically, 6Hz pacing caused Model rat hearts to increase APD90 (Sham = 31.45 ± 1.91 vs. Model = 70.50 ± 6.73, P < 0.01), rise time (Sham = 12.48 ± 0.50 vs. Model = 25.07 ± 3.40, P < 0.01), and IQR (Sham = 4.052 ± 1.99814 vs. Model = 27.426 ± 6.06782, P < 0.01), which was reduced after treatment with WK-L (57.68 ± 5.54, P < 0.05; 16.35 ± 1.00, P < 0.01; 14.94 ± 3.011, P < 0.01), WK-M (55.38 ± 3.67, P < 0.01; 16.37 ± 0.99, P < 0.01; 10.57 ± 2.38, P < 0.01), WK-H (52.24 ± 4.68, P < 0.01; 15.13 ± 0.46, P < 0.01; 7.03 ± 1.70, P < 0.01), and Met (56.98 ± 9.10, P < 0.05; 16.20 ± 1.21, P < 0.01; 9.42 ± 1.39, P < 0.01), respectively. Compared to that of Sham APD30/80 (Sham = 0.50 ± 0.02 vs. Model = 0.33 ± 0.01, P < 0.01) and the velocity (Sham = 10.12 ± 2.13 vs. Model = 3.13 ± 0.23, P < 0.05) changed slower were both significantly reduced in the Model. In contrast, WK or Met-treatment increased the APD30/80 (WK-L = 0.38 ± 0.01, P < 0.01; WK-M = 0.39 ± 0.01, P < 0.01; WK-H = 0.40 ± 0.02, P < 0.01; and Met = 0.40 ± 0.01, P < 0.01) and velocity (WK-L = 4.54 ± 1.11, P > 0.05; WK-M = 4.62 ± 0.58, P > 0.05; WK-H = 4.52 ± 0.49, P > 0.05 and Met = 4.44 ± 0.47, P > 0.05) in **Figure 2C-F**.

AF was more likely to happen when the cycle duration (S1S1 is 100-50 ms) was shortened. The APD alternans sequence changed unevenly across the atrium, with one area displaying a greater APD alternans sequence amplitude and three sections displaying opposing small-large sequences. Space inconsistency of APD alternans in the Model stood out the most among them. The incidence of spatial incongruent alternations in ISIAF rats were significantly higher at 90-50 ms than in Sham rats (90 ms: Sham = 0.055 ± 0.03 vs. Model = 0.18 ± 0.05, P < 0.05; 80ms: Sham = 0.14 ± 0.07 vs. Model = 0.33 ± 0.05, P < 0.01; 70ms: Sham = 0.10 ± 0.04 vs. Model = 0.44 ± 0.10, P < 0.01; 60ms: Sham = 0.10 ± 0.06 vs. Model = 0.60 ± 0.06, P < 0.01; 50ms: Sham = 0.14 ± 0.03 vs. Model = 0.70 ± 0.05, P < 0.01), but was suppressed at 80-50 ms after treatment with WK-L (80ms: 0.16 ± 0.04, P < 0.01; 70ms: 0.34 ± 0.03, P > 0.05; 60ms: 0.42 ± 0.06, P < 0.01; 50ms: 0.56 ± 0.03, P < 0.01), WK-M (80ms: 0.15 ± 0.09, P < 0.01; 70ms: 0.34 ± 0.13, P > 0.05; 60ms: 0.41 ± 0.14, P < 0.01; 50ms: 0.47 ± 0.05, P < 0.01),WK-H (80ms: 0.08 ± 7.56E-04, P < 0.01; 70ms: 0.21 ± 0.06, P < 0.01; 60ms: 0.23 ± 0.12, P < 0.01; 50ms: 0.38 ± 0.07, P < 0.01) or Met (80ms: 0.10 ± 0.03, P < 0.01; 70ms: 0.12 ± 0.02, P < 0.01; 60ms: 0.14 ± 0.08, P < 0.01; 50ms: 0.12 ± 0.02, P < 0.01) groups (**Figure 2G**).

### Ischemic stroke-induced CaT alterations that were recovered by WK

CaT undergoes several repolarization phases, such as priming, outbreak, and decay, and it is regulated by intracellular calcium signaling. Ischemic stroke-induced CaT changes in the whole isolated heart with sinus rhythm. Compared with the Model, the treatment of WK-H (4.43 ± 1.48, P < 0.05) had an improved effect, and the velocity of the Model was altered compared to the Sham (Sham = 8.25 ± 1.74 vs. Model = 2.01 ± 0.18, P < 0.01, **Supplemental Figure 3A**). The Model rat hearts facilitated the CaT90 protraction (Sham = 54.84 ± 2.75 vs. Model = 105.92 ± 9.06, P < 0.01) while WK-L (82.95 ± 11.00, P < 0.01), WK-M (70.75 ± 3.95, P < 0.01), WK-H (64.23 ± 3.31, P < 0.01) and Met (60.57 ± 6.91, P < 0.01) treatment effectively alleviated the CaT90 extension (**Supplemental Figure 3C**). Ischemic stroke also resulted in a longer rise time (Sham = 17.25 ± 0.81 vs. Model = 27.02 ± 2.52, P < 0.01) and increased IQR heterogeneity (Sham = 9.23 ± 2.86 vs. Model = 25.79 ± 3.79, P < 0.01). WK or Met effectively shortened the delayed rise time (WK-L = 18.52 ± 1.61, P < 0.01; WK-M = 18.77 ± 0.92, P < 0.01; WK-H = 16.89 ± 2.50, P < 0.01; and Met = 17.30 ± 2.06, P < 0.01) and attenuated IQR heterogeneity values (WK-L= 16.38 ± 1.92, P < 0.01; WK-M = 10.81 ± 2.35, P < 0.01; WK-H = 10.21 ± 1.06, P < 0.01; and Met = 15.05 ± 0.83, P < 0.01) when compared to the Model (**Supplemental Figure 3B and D**).

Atrial conduction maps of Ca^2+^ and atrial CaT90 maps (6Hz) (**Figure 2H-I**) indicated that cerebral ischemia caused clear CaT90 extension, which was improved by Met or WK. Meanwhile, 6Hz pacing increased CaT90 (Sham = 45.26 ± 3.07 vs. Model = 90.82 ± 9.61, P < 0.01), rise time (Sham = 15.10 ± 0.55 vs. Model = 23.68 ± 1.02, P < 0.01), and IQR (Sham = 7.89 ± 0.84 vs. Model = 27.75 ± 7.30, P < 0.01) in the Model, which was reduced by treatment with WK-L (68.17 ± 9.94, P < 0.01; 17.25 ± 1.74, P < 0.01; and 19.53 ± 3.66, P > 0.05), WK-M (60.05 ± 7.38, P < 0.01; 16.44 ± 1.87, P < 0.01; and 10.98 ± 1.40, P < 0.01), WK-H (59.34 ± 5.19, P < 0.01; 16.68 ± 1.62, P < 0.01; and 10.45 ± 1.62, P < 0.01), and Met (60.56 ± 2.37, P < 0.01; 18.98 ± 0.25, P < 0.01; and 14.13 ± 1.27, P < 0.01), respectively. Compared to Sham, the Model had slower velocity change (Sham= 10.25 ± 1.21 vs. Model= 2.38 ± 0.35, P < 0.05). In contrast, WK-L, WK-M, WK-H, or Met treatments increased the velocity (WK-L=3.27 ± 0.12, P > 0.05; WK-M=3.37 ± 0.32, P > 0.05; WK-H=4.55 ± 0.94, P < 0.01; Met=3.21 ± 0.32, P > 0.05, **Figure 2J-L)**. The four heat mapping sites chosen by APD and CaT alternating, along with the computation process, were displayed in **Figure 2M**.

Atrial alternans were induced by incrementally decreasing cycle lengths (100-50 ms). **Figure 2N** shows the representative heat maps of CaT alternans magnitude from 100 ms to 50 ms. Throughout the atrium, the CaT alternans sequence varied unevenly, with three areas showing opposing small-large sequences and one portion showing a larger CaT alternans sequence amplitude. Compared to the Sham, the Model had a greater CaT alternans ratio from 90 ms to 50 ms (90ms: Sham = 0.06 ± 0.01 vs. Model = 0.16 ± 0.06, P < 0.05; 80ms: Sham = 0.05 ± 0.04 vs. Model = 0.34 ± 0.02, P < 0.01; 70ms: Sham = 0.02 ± 0.01 vs. Model = 0.51 ± 0.08, P < 0.01; 60ms: Sham = 0.06 ± 0.04 vs. Model = 0.68 ± 0.04, P < 0.01; 50ms: Sham = 0.05 ± 0.05 vs. Model = 0.76 ± 0.04, P < 0.01). However, the WK-L (80ms: 0.33 ± 0.05, P > 0.05, 70ms: 0.39 ± 0.06, P < 0.05; 60ms: 0.48 ± 0.03, P < 0.01; 50ms: 0.58 ± 0.06, P < 0.01), WK-M (80ms: 0.11 ± 0.05, P < 0.01; 70ms: 0.24 ± 0.02, P < 0.01; 60ms: 0.30 ± 0.03, P < 0.01; 50ms: 0.46 ± 0.04, P < 0.01), WK-H (80ms: 0.23 ± 0.10, P < 0.01; 70ms: 0.27 ± 0.07, P < 0.01; 60ms: 0.30 ± 0.08, P < 0.01; 50ms: 0.38 ± 0.05, P < 0.01), or Met (80ms: 0.06 ± 0.04, P < 0.01; 70ms: 0.12 ± 0.09, P < 0.01; 60ms: 0.10 ± 0.07, P < 0.01; 50ms: 0.32 ± 0.02, P < 0.01) treatment partially alleviated the CaT alternans ratios, particularly from 80 ms to 50 ms (**Figure 2N**).

**Figure 3:**
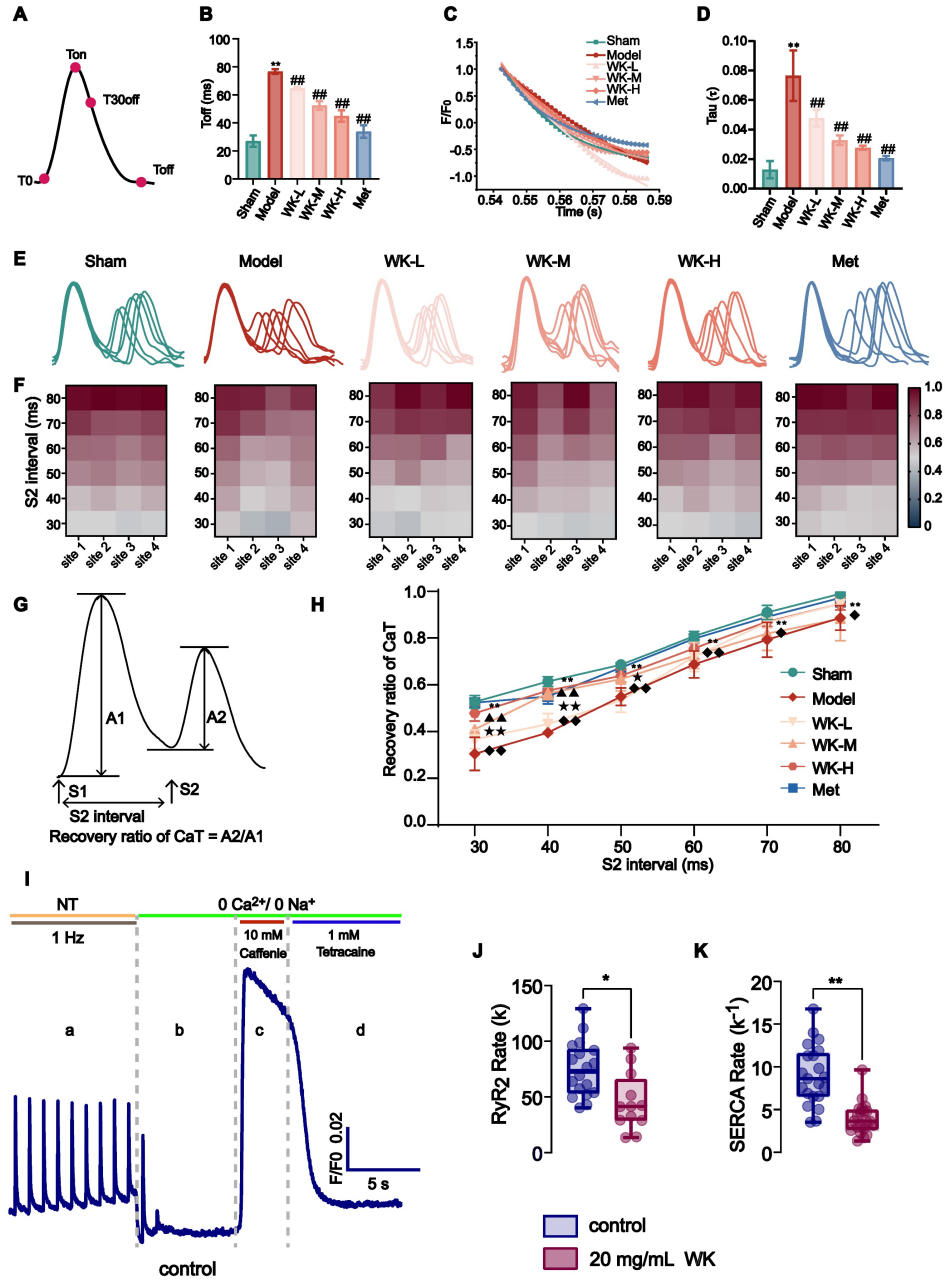
** AF-related changes and Ca^2+^ release restitution in the ISIAF model with or without WK treatment.** (A, B) Toff calculation diagram and its values under each condition. (C, D) Typical examples of Tau and calculation of Tau under each condition. Values were expressed as mean ± SD (n=3-5, ^*^P < 0.05 and ^**^P < 0.01, Model vs. Sham; ^#^P < 0.05 and ^##^P < 0.01, Model vs. WK-L) (E) Representative recordings of cytosolic CaTs at various S1S2 coupling intervals. (F) The heat maps of different conduction rates of the six groups. (G) Calculation diagram of recovery ratio of CaT. (H) Depiction of recovery ratio of CaT at the S1S2 integral level. Values were expressed as mean ± SD (n=3-5, ^*^P < 0.05 and ^**^P < 0.01, Model vs. Sham; ^#^P < 0.05 and ^##^P < 0.01, Model vs. WK-L; ^▲^P < 0.05 and ^▲▲^P < 0.01, Model vs. WK-M; ^★^P < 0.05 and ^★★^P < 0.01, Model vs. WK-H; ^◆^P < 0.05 and ^◆◆^P < 0.01, Model vs. Met). (I) Graphical representation of SR Ca^2+^ fluxes estimation protocol [a: basic Ca^2+^ transient parameters, b: RyR Ca^2+^ leak, c: SR Ca^2+^ content, d: SERCA activity; NT, normal Tyrode solution. (J, K) Quantification of RyR_2_ and SERCA functions of rate-constants. [control n=6/18 (animals/cells), WK n=7/20 (animals/cells); ^*^P < 0.05 and ^**^P < 0.01, Sham vs. WK].

T0, Ton, T30off, and Toff marked each transition point (**Figure 3A**). The recovery time of Ca^2+^ from Ton to Toff is shown in **Figure 3B**. A 6Hz pacing-induced ISIAF rat hearts to increase the steepness of CaT restitution (Sham = 27.04 ± 4.01 vs. 76.68 ± 1.68, P < 0.01) that was reduced after treatment with WK-L, WK-M, WK-H and Met to 64.77 ± 0.89 (P < 0.01), 52.44 ± 3.10 (P < 0.01), 44.98 ± 4.08 (P < 0.01) and 33.90 ± 4.43 (P < 0.01). A typical CaT restitution curve fitted by monoexponential relationships is shown in **Figure 3C**. AF increased the time constant of intracellular Ca^2+^ decay (Tau) in the Model (Sham = 0.01 ± 0.01 vs 0.08 ± 0.02, P < 0.01). WK-L (0.05 ± 0.01, P < 0.01), WK-M (0.03 ± 0.01, P < 0.01), WK-H (0.03 ± 0.01, P < 0.01), and Met (0.02 ± 0.01, P < 0.01) reduced the recovery of Tau (**Figure 3A-D**). The calculation of Ton and Tau was also discussed in the previous literature 50,51.

The refractory duration of CaT is crucial for the development of both CaT and APD alternans 40,41. An S1S2 procedure was utilized to measure CaT recovery from refractoriness (A2/A1 at each S2 interval). Cardiomyocytes were subjected to an S1 stimulation, which caused an intracellular Ca^2+^ release. An additional S2 stimulation could significantly increase the next Ca^2+^ transient's amplitude in a steady state. The ratio of the intracellular Ca^2+^ transient caused by an S1 impulse to the one caused by an S2 impulse is known as CaT refractoriness (also known as RyR_2_ refractoriness) 40. Representative CaT recordings from each group at various S1S2 coupling intervals are shown in **Figure 3E**. Representative heat maps of the CaT recovery ratio at 4 atrial sites are shown in **Figure 3F** and the recovery ratio of CaT was calculated in **Figure 3G**. The recovery of CaT at the entire atrium level is shown in **Figure 3H**. Compared to the Sham, the Model rats had a greater CaT alternans ratio from 80 ms to 30 ms (80ms: Sham = 0.98 ± 0.03 vs. Model = 0.89 ± 0.05, P < 0.01; 70ms: Sham = 0.91 ± 0.03 vs. Model = 0.79 ± 0.07, P < 0.01; 60ms: Sham = 0.80 ± 0.03 vs. Model = 0.69 ± 0.06, P < 0.01; 50ms: Sham = 0.66 ± 0.07 vs. Model = 0.55 ± 0.04, P < 0.01; 40ms: Sham = 0.57 ± 0.10 vs. Model = 0.40 ± 0.02, P < 0.01; 30ms: Sham = 0.48 ± 0.10 vs. Model = 0.30 ± 0.07, P < 0.01). The CaT recovery with the various S2 intervals was much higher from 80 ms-30 ms in the WK-L (80ms: 0.95 ± 0.05, P > 0.05; 70ms: 0.87 ± 0.02, P > 0.05; 60ms: 0.72 ± 0.06, P > 0.05; 50ms: 0.53 ± 0.05, P > 0.05; 40ms: 0.43 ± 0.05, P > 0.05; 30ms: 0.37 ± 0.04, P > 0.05), WK-M (80ms: 0.88 ± 0.09, 70ms: 0.82 ± 0.07, 60ms: 0.72 ± 0.05, 50ms: 0.62 ± 0.05, 40ms: 0.56 ± 0.02, P < 0.01; 30ms: 0.41 ± 0.01, P < 0.01), WK-H (80ms: 0.95 ± 0.03, P > 0.05; 70ms: 0.87 ± 0.02, P > 0.05; 60ms: 0.76, P > 0.05; 50ms: 0.64 ± 0.03, P < 0.05; 40ms: 0.58 ± 0.03, P < 0.01; 30ms: 0.48 ± 0.03, P < 0.01), and Met (80ms: 0.97 ± 0.04, P < 0.05; 70ms: 0.89 ± 0.01, P < 0.05; 60ms: 0.80 ± 0.01, P < 0.01; 50ms: 0.67 ± 0.02, P < 0.01; 40ms: 0.55 ± 0.03, P < 0.01; 30ms: 0.52 ± 0.01, P < 0.01) groups compared with the Model group, the WK and Met groups only exhibited partial effects. Our single-cell calcium imaging complements tissue optical mapping. To evaluate the impact of WK on the functions of RyR_2_ and SERCA, we employed a specialized protocol designed to differentiate various calcium release and uptake mechanisms (Figure 3I). This analysis revealed significant differences in Ca^2+^ handling by atrial cardiomyocytes, evidenced by a reduced slope constant for both RyR_2_ (RyR_2_ rate = 45.33 ± 25.71) and SERCA (SERCA rate ^-1^ = 3.95 ± 1.86) in the WK group compared to the control group (RyR_2_ rate = 74.90 ± 24.75 and SERCA rate ^-1^ = 8.97 ± 3.50, P < 0.01 or P < 0.05). These findings indicated a slower release by RyR_2_ and a slower uptake by SERCA in the WK group. The reduced slope constant of RyR_2_ observed in single-cell calcium imaging within the WK group, in conjunction with the rise time data from tissue optical mapping, suggested a diminished RyR_2_ release under WK treatment (Figure 3J). Similarly, the decreased slope constant of SERCA in single-cell calcium imaging, when considered alongside the Tau value from tissue optical mapping, indicates a decline in the functional recovery of SERCA (Figure 3K). The incidence of arrhythmia in ischemic stroke rats was significantly increased by optical mapping. Moreover, through the judgment of sinus rhythm in a natural state, and then through fixed frequency stimulation of 6Hz, and gradually high-frequency stimulation of S1S2 and S1S1, the conduction time, rise time, and IQR of AP and Ca were proved to be significantly different in electrophysiological indexes of ischemic stroke rats compared with Sham group. Therefore, we conclude that the rat model of ischemic stroke-induced AF (ISIAF) is valid.

### Identification of atrium genes altered by ISIAF and restored by WK

**Figure 4:**
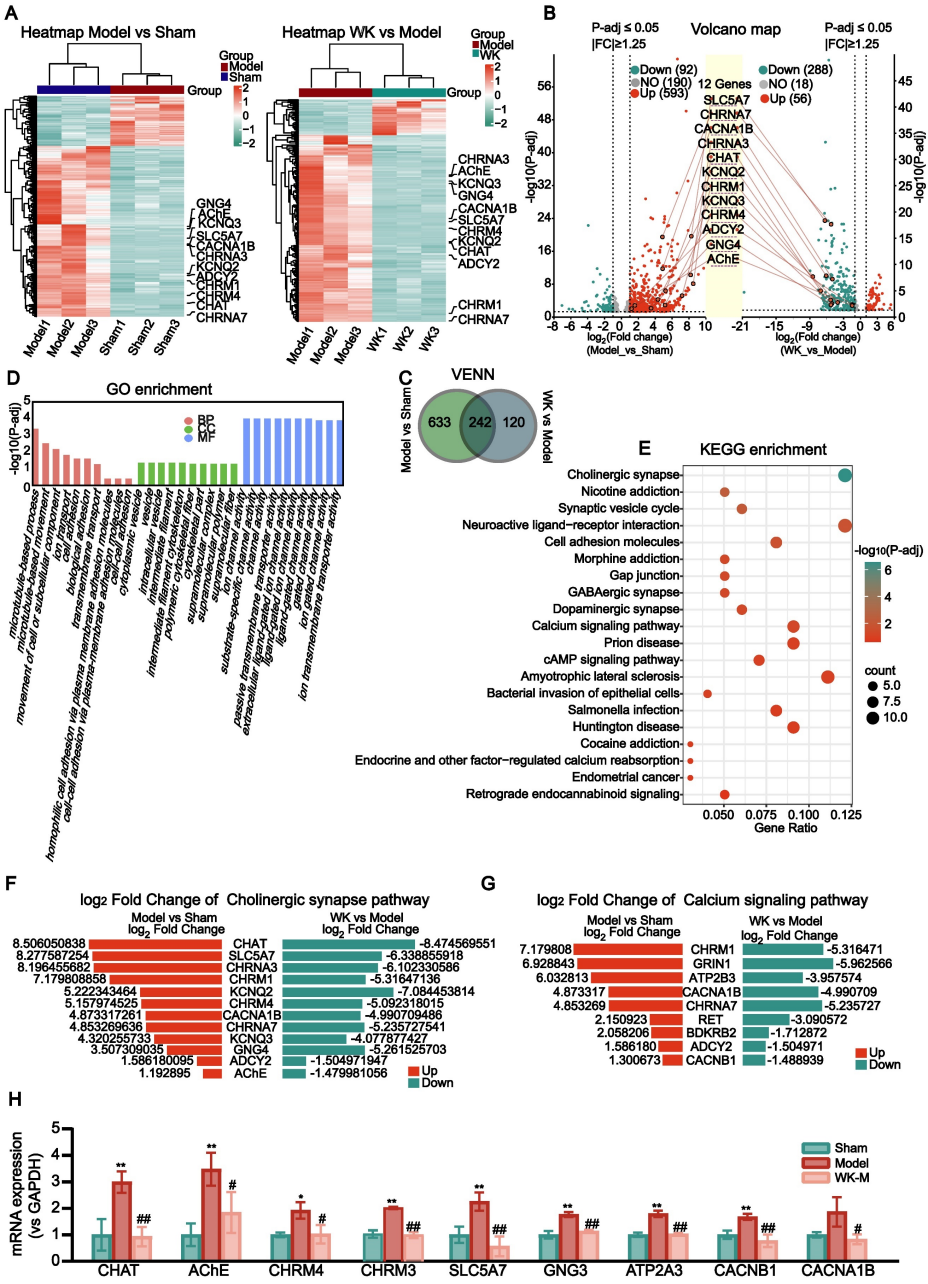
** Transcriptome sequencing and IPA analysis of rat atrium in ISIAF and WK treatment.** (A) Hierarchical clustering of the Sham, Model, and WK-M groups. The 12 genes for the cholinergic synaptic signaling pathway are labeled. Left panel: Model vs Sham, right panel: WK vs Model. (B) A volcano graph showing altered gene distributions in the Sham, Model, and WK-M groups (|fold change| ≥ 1.25 and P-adj ≤ 0.05). (C) A Venn diagram showing altered genes in ISIAF (Model vs Sham) and after WK treatment (WK vs Model). (D) GO enrichment analysis ranked ion channel activity the highest among the top 10 functions. (E) IPA Core analysis identified the cholinergic synaptic signaling pathway as the top 1 altered pathway associated with disease. The fold change and significance level of the difference in the gene expression were represented by the abscissa and ordinate, respectively. The 12 genes of the cholinergic synaptic signaling pathway were labeled. Genes that have increased or decreased in expression were shown by red or green dots, respectively. There were three independent animals in each group (n = 3). (F) Log_2_Fold Change values of the genes in the cholinergic synaptic signaling pathway. (G) Log_2_Fold Change values of the genes in the calcium signaling pathway. (H) RT-PCR verification of WK-M regulated genes in cholinergic and calcium signaling pathway. The mRNA expression levels of the CHAT, AChE, CHRM4, CHRM3, SLC5A7, GNG3, ATP2A3, CACNB1 and CACNA1B. Values are given as mean ± SD (n = 3, ^*^P < 0.05 and ^**^P < 0.01 Model vs. Sham, ^#^P < 0.05 and ^##^P < 0.01 Model vs. WK).

The atrium tissues of the Sham, Model, and WK-treated rats were subjected to RNA-seq analysis to identify the differentially expressed genes (DEGs) in the atrium after ischemic stroke as well as those regulated by WK (**Figure 4A**). Among the 875 DEGs altered by ISIAF, 242 genes were regulated by WK, indicating that these genes may be responsible for WK atrium action after ischemic stroke (**Figure 4C**). Core analysis and GO enrichment of the 242 WK-regulated DEGs in the atrium tissue of ISIAF rats indicated that ion channel and transport-related processes are among the top biological processes (BP), molecular functions (MF), and cellular components (CC) (**Figure 4D**). Core analysis and KEGG enrichment analysis indicated that cholinergic synaptic signaling ranked first among the canonical pathways impacted by WK in ISIAF rat atrium, which were sorted in descending order based on the -log(P-adj) score (**Figure 4E**). The other top canonical pathways affected by WK in ISIAF included nicotine addiction synaptic vesicle cycle, neuroactive ligand-receptor interaction, cell adhesion molecules, gap junction, GABAergic synapse, morphine addiction, dopaminergic synapse, amyotrophic lateral sclerosis, cAMP signaling pathway, prion disease, bacterial invasion of epithelial cells, cocaine addiction, salmonella infection, Huntington disease, endocrine and other factor-regulated calcium reabsorption, endometrial cancer, and retrograde endocannabinoid signaling (**Figure 4E**). As shown in **Figure 4E**, among the 875 genes differentially expressed in Model vs. Sham and the 362 genes differentially expressed in WK vs. Model, most cholinergic synaptic signaling genes were reciprocally regulated. **Figure 4F-G** showed the log_2_Fold Change values of the 12 genes in the cholinergic synaptic signaling pathway and 9 genes in the calcium signaling pathway, comparing both the Model vs. Sham and WK vs. Model groups. A schematic diagram of the mechanism of WK ameliorated ISIAF via regulating cholinergic and calcium signaling pathways is presented in **Figure 10**.

### Validation of cholinergic and calcium signaling gene alteration in ISIAF rat atrium

The transcriptome analysis indicated that the most significantly altered genes in ISIAF and reversed by WK are in cholinergic and calcium signaling. SLC6A15, SYT4, INA, and CHRM1 are from the RNA-seq validation transcriptome. CHAT, AChE, CHRM4, SLC5A7, CACNB1, and CACNA1B are derived from RNA-seq validation cholinergic and calcium signaling. CHRM3, GNG3, and ATP2A3 were verified by RT-PCR. RT-PCR experiment confirmed that compared with the Sham group, the mRNA expression levels of SLC6A15 (Sham = 1.00 ± 0.37 vs. Model = 2.32 ± 0.18, P < 0.01), SYT4 (Sham = 1.00 ± 0.19 vs. Model = 1.67 ± 0.45, P > 0.05), INA (Sham = 1.00 ± 0.64 vs. Model = 2.97 ± 0.74, P < 0.01) and CHRM1 (Sham = 1.00 ± 0.48 vs. Model = 4.16 ± 1.32, P < 0.01) were up-regulated. Compared with the Model, WK-M significantly down-regulated mRNA expression levels of SLC6A15 (1.33 ± 0.12, P < 0.01), SYT4 (0.84 ± 0.09, P < 0.05), INA (0.69 ± 0.43, P < 0.01), and CHRM1 (1.17 ± 0.36, P < 0.01) (**Supplemental Figure 4A-E**). RT-PCR also verified that CHAT (Sham = 1.00 ± 0.60 vs. Model = 3.00 ± 0.41, P < 0.01), SLC5A7 (Sham = 1.00 ± 0.31 vs. Model = 2.25 ± 0.34), CHRM4 (Sham = 1.00 ± 0.091 vs. Model = 1.92 ± 0.31, P < 0.05), CACNA1B (Sham = 1.00 ± 0.10 vs. Model = 1.87 ± 0.56, P > 0.05), AChE (Sham = 1.00 ± 0.43 vs. Model = 3.48 ± 0.62, P < 0.01), CACNB1 (Sham = 1.00 ± 0.16 vs. Model = 1.67 ± 0.12, P < 0.01), ATP2A3 (Sham = 1.00 ± 0.08 vs. Model = 1.80 ± 0.12, P < 0.01), GNG3 (Sham = 1.00 ± 0.14 vs. Model = 1.77 ± 0.09, P < 0.01) and CHRM3 (Sham = 1.00 ± 0.14 vs. Model = 1.95 ± 0.04, P < 0.01) genes regulated in cholinergic signaling pathway and calcium signaling pathway were significantly up-regulated in Model group compared with the Sham. Compared with the Model, CHAT (0.93 ± 0.37, P < 0.01), SLC5A7 (0.57 ± 0.37, P < 0.01), CHRM4 (1.02 ± 0.35, P < 0.05), CACNA1B (0.83 ± 0.19, P < 0.05), AChE (1.84 ± 0.77, P < 0.05), CACNB1 (0.77 ± 0.23, P < 0.01), ATP2A3 (1.02 ± 0.05, P < 0.01), GNG3 (1.13 ± 0.06, P < 0.01) and CHRM3 (0.96 ± 0.11, P < 0.01) genes regulated in WK-M were significantly down-regulated (**Figure 4J-K**).

### Effects of WK on I_Ca-L_

**Figure 5:**
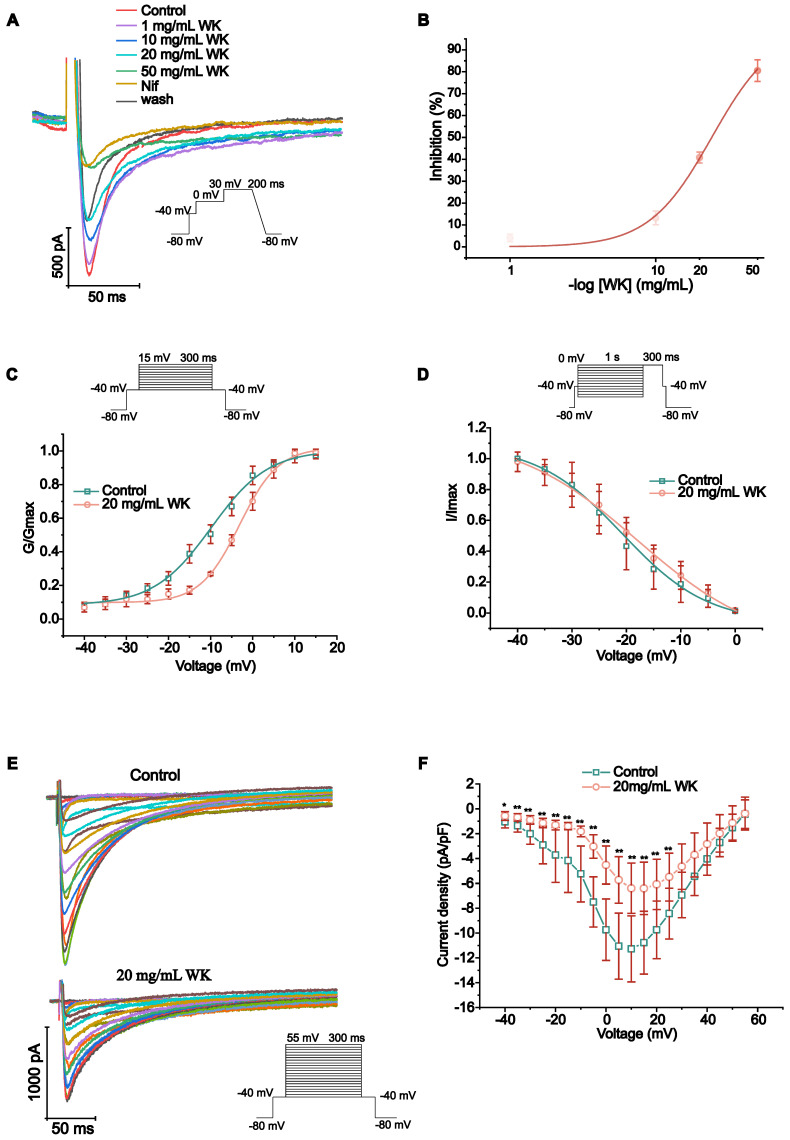
** Verification of WK regulation of the L-type Ca^2+^ channel dynamics.** Calcium voltage-gated channel auxiliary subunit beta 1 (CACNB1) constitutes the beta subunit of the L-type calcium channel. (A) Representative trace of the L-type Ca^2+^-current (I_Ca-L_) recorded in Control, WK (1, 10, 20, 50 mg/mL), Nif and wash. (B) The concentration-response curve representing the percentage of inhibition of WK (n=3-5). (C) Standardized steady-state activation of I_Ca-L_ under Control and 20 mg/mL WK. (D) Standardized steady-state deactivation of I_Ca-L_ under Control and 20 mg/mL WK treatment (n=6). (E, F) Sample trace and pooled data showed the effect of I-V relationship under Control and 20 mg/mL WK treatment. Values are given as mean ± SD (n = 6, ^#^P < 0.05 and ^##^P < 0.01 WK vs. Control).

The peak of I_Ca-L_ may be greatly inhibited by WK, according to **Figure 5A-F**. The effects of various WK concentrations on I_Ca-L_ were shown. I_Ca-L_ was gradually restricted when WK concentrations (1, 10, 20, 50 mg/mL, or Nif) were raised. According to the typical trajectory of the point in **Figure 5A**, WK reduced I_Ca-L_ in a concentration-dependent manner. The WK's half-maximal inhibitory concentration (IC_50_) is 24.2546 ± 2.0517 mg/mL. WK dose-dependently lowered I_Ca-L_ by 4.072 ± 1.756 %, 13.225 ± 3.163 %, 40.819 ± 2.509 % and 80.512 ± 4.926 % at 1, 10, 20, and 50 mg/mL, respectively (**Figure 5B).**

The steady-state activation voltage and I_Ca-L_ inactivation were shown to be voltage-dependent in Figure 5F-G. According to the preceding article, the statistical formula for the activation, deactivation, and IC_50_ curves were as follows 52. The value of V_1/2_ of activated I_Ca-L_ in the Control group was -9.831 ± 0.631 mV. Nevertheless, the values of V_1/2_ for activation with WK 20 mg/mL were -3.140 ± 0.440 mV, respectively (P < 0.01 or P < 0.05, **Figure 5C**). The value of V_1/2_ of the steady-state inactivation was -20.786 ± 0.615 mV in the Control group. In the presence of WK 20 mg/mL, the values of V_1/2_ for inactivation were -17.981 ± 0.913 mV, respectively (P < 0.01 or P < 0.05, **Figure 5D**). At -40mV, the pulse of 20ms inactivated the Na^+^ channel. 300ms depolarized pulses were used in 5mV increments to obtain I_Ca-L_ of atrial myocytes at different voltages from -40mV to 55mV. Plot the test voltage as the X axis, current density (pA/pF) as the Y axis, and Control and WK 20 mg/mL using the I-V relationship. The I-V curves indicated an increasing trend. Activation starts from -30mV, peak potential is 10 mV, and reverse potential is +55 mV. After infusion with extracellular solution containing WK (20 mg/mL), the peak current density of I_Ca-L_ activation decreased in the voltage range of -40 mV ~ 55 mV. From (-11.277 ± 2.663 pA/pF) to (-6.394 ± 2.023 pA/pF), the I-V curve shifted upward, but the activation potential, peak potential, reversal potential, and curve shape did not change (P < 0.01 or P < 0.05, **Figure 5E-F**).

### Identification of WK active ingredients that interact with CACNB1 and CHRM3

**Figure 6:**
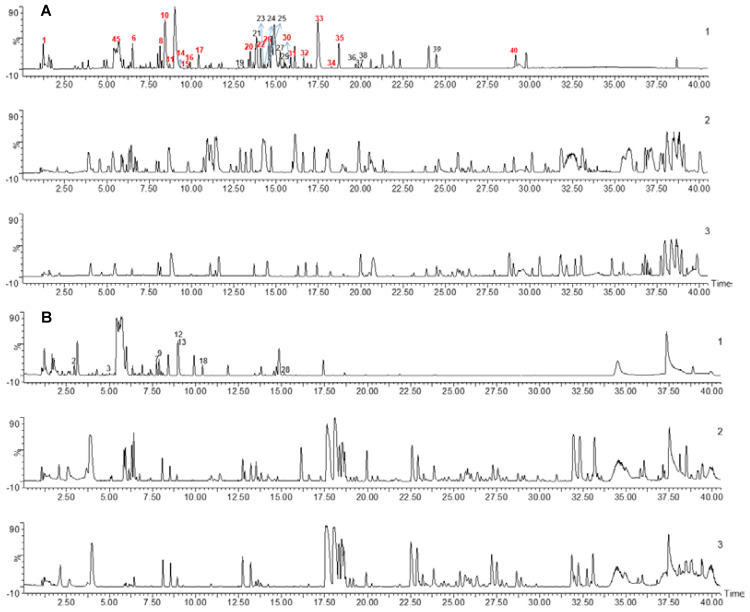
** Chemical profiling of WK active ingredients in rat plasma.** UPLC-MS base peak intensity chromatograms in negative (A) or positive (B) modes. In each panel, WK aqueous solution (Top, 1) Plasma after 1h WK administration (Middle, 2) and Blank plasma (Bottom, 3) are shown.

To detect WK active ingredients that enter the rat plasma, WK aqueous solution, rat blank plasma, and WK-containing plasma samples were analyzed. The base peak chromatogram of WK aqueous solution, rat blank plasma, and drug-containing plasma samples in positive and negative ion modes are shown in **Figure 6**. A total of 40 chemical components were found in WK samples, mostly sugars and saponins (**Table 1**). Through a comprehensive analysis of the basic peak ion diagram of rat blank plasma and WK-containing plasma and the components of WK water extract, 21 components were identified that enter the blood, mainly including sugars, saponins, and flavonoids, as summarized in **Table 1**.

**Figure 7:**
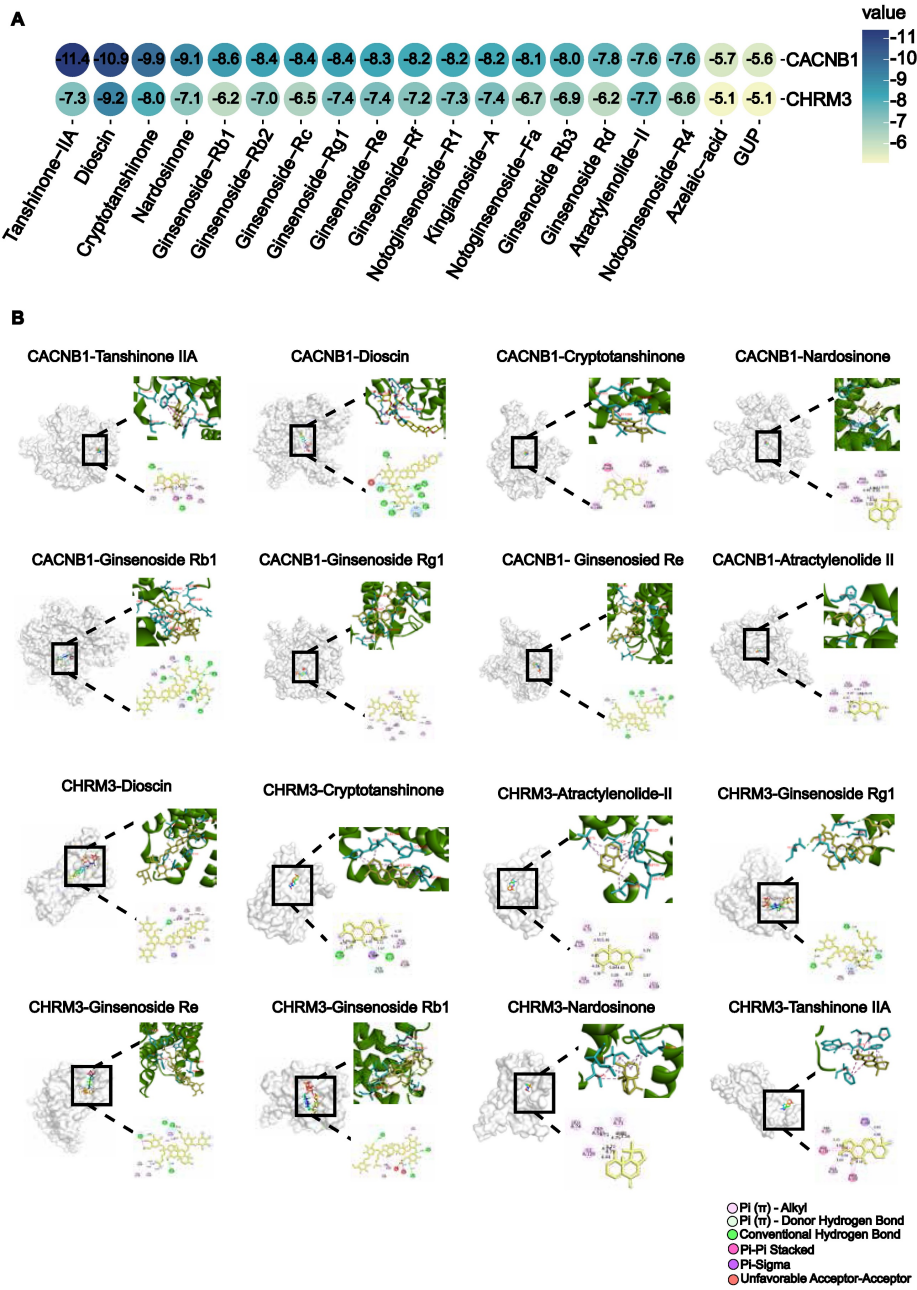
** Virtual screen of WK active compounds targeting muscarinic acetylcholine receptor M3 (CHRM3) and calcium voltage-gated channel auxiliary subunit beta 1 (CACNB1) by molecular docking.** (A) The heatmap of docking scores of 19 WK active compounds binding to key targets CACNB1 and CHRM3 (-CDOCKER ENERGY score, kcal/mol). In the bubble plot, the binding energies were represented by bubble colors. A lower stability value indicates a more stable complex. (B) Representative docking complex of key targets and corresponding compounds.

To determine which active compounds in WK interact with CACNB1 and CHRM3, the UPLC/Q-TOF-MS detected WK ingredients were screened by molecular docking. The top 5 WK compounds with the highest affinity to interact with CACNB1 (-CDOCKER ENERGY score) were Tanshinone-IIA, Dioscin, Crypeotanshinone, Nardosinone and Ginsenoside Rb1 (**Figure 7A**). The top 5 WK compounds with the highest affinity to interact with CHRM3 (-CDOCKER ENERGY score) were Dioscin, Crypeotanshinone, Ginsenoside Rg1, Ginsenoside Re and Tanshinone-IIA (**Figure 7A**). WK compounds-CACNB1 and WK compounds-CHRM3 were depicted by active binding site (3D & 2D model, **Figure 7B**). Of the 21 components of WK entering the blood, Ditertbutyl phthalate was a plasticizer, and the structure of Hexyl-D-glucopyranosyl-(1-2)-D-glucopyranoside was unavailable.

### Effects of active compounds of WK on I_Ca-L_

**Figure 8:**
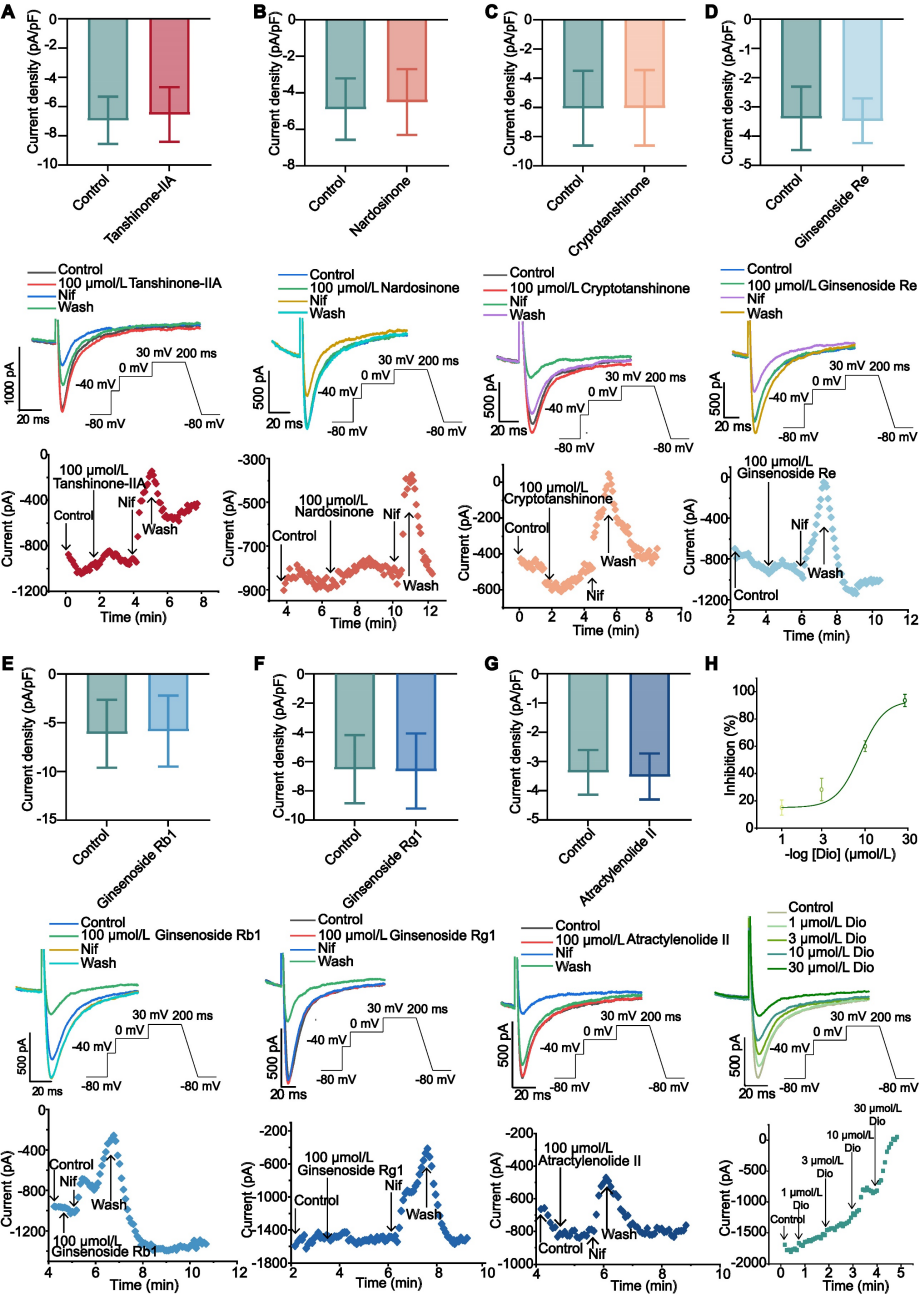
** The effect of WK active compounds on I_Ca-L_ in rat atrium myocytes.** (A-H) Whole cell recordings showing (top to bottom) current density, sample trace, combined data and process time of I_Ca-L_ recorded during exposure to (A) Tanshinone-IIA (100 μmol/L), (B) Nardosinone (100 μmol/L), (C) Cryptotanshinone (100 μmol/L), (D) Ginsenoside Re (100 μmol/L), (E) Ginsenoside Rb1 (100 μmol/L), (F) Ginsenoside Rg1 (100 μmol/L), (G) Atractylenolide (100 μmol/L) and washing under Control conditions. The data are expressed as Mean ± SD (n=3-4). Compared with Control, P > 0.05. (H) The inhibition curve (top), an example trace (middle), time course (bottom) of I_Ca-L_ recorded under Control conditions of 1, 3, 10, and 30 μmol/L Dioscin. Concentration-response curve representing the percentage of inhibition of Dioscin (n=4).

Ginsenoside Rb1 (10, 50 and 100 µmol/L), Ginsenoside Re (10, 50 and 100 µmol/L), Ginsenoside Rg1 (10, 50 and 100 µmol/L), Atractylenolide II (10, 50 and 100 µmol/L), Tanshinone-IIA (10, 50 and 100 µmol/L), Cryptotanshinone (10, 50 and 100 µmol/L) and Nardosinone (10, 50 and 100 µmol/L) had no obvious inhibitory effect on I_Ca-L_ (P > 0.05, **Figure 8A-G** and **Supplemental Figure 5A-B**). I_Ca-L_ was gradually restricted when Dioscin concentrations (1, 3, 10, 30 µmol/L) were raised. According to the typical trajectory of the point in **Figure D-E**, Dioscin reduces I_Ca-L_ in a concentration-dependent manner. The Dioscin's half-maximal inhibitory concentration (IC_50_) is 8.666 ± 0.777 µmol/L. Dioscin dose-dependently lowered I_Ca-L_ by 15.065 ± 5.513 %, 28.270 ± 8.184 %, 60.040 ± 3.987 %, and 93.574 ± 4.527 % at 1, 3, 10, and 30 µmol/L, respectively (**Figure 8H**).

### Effect of WK and its active compounds on Ach-activated HL-1 cells

**Figure 9:**
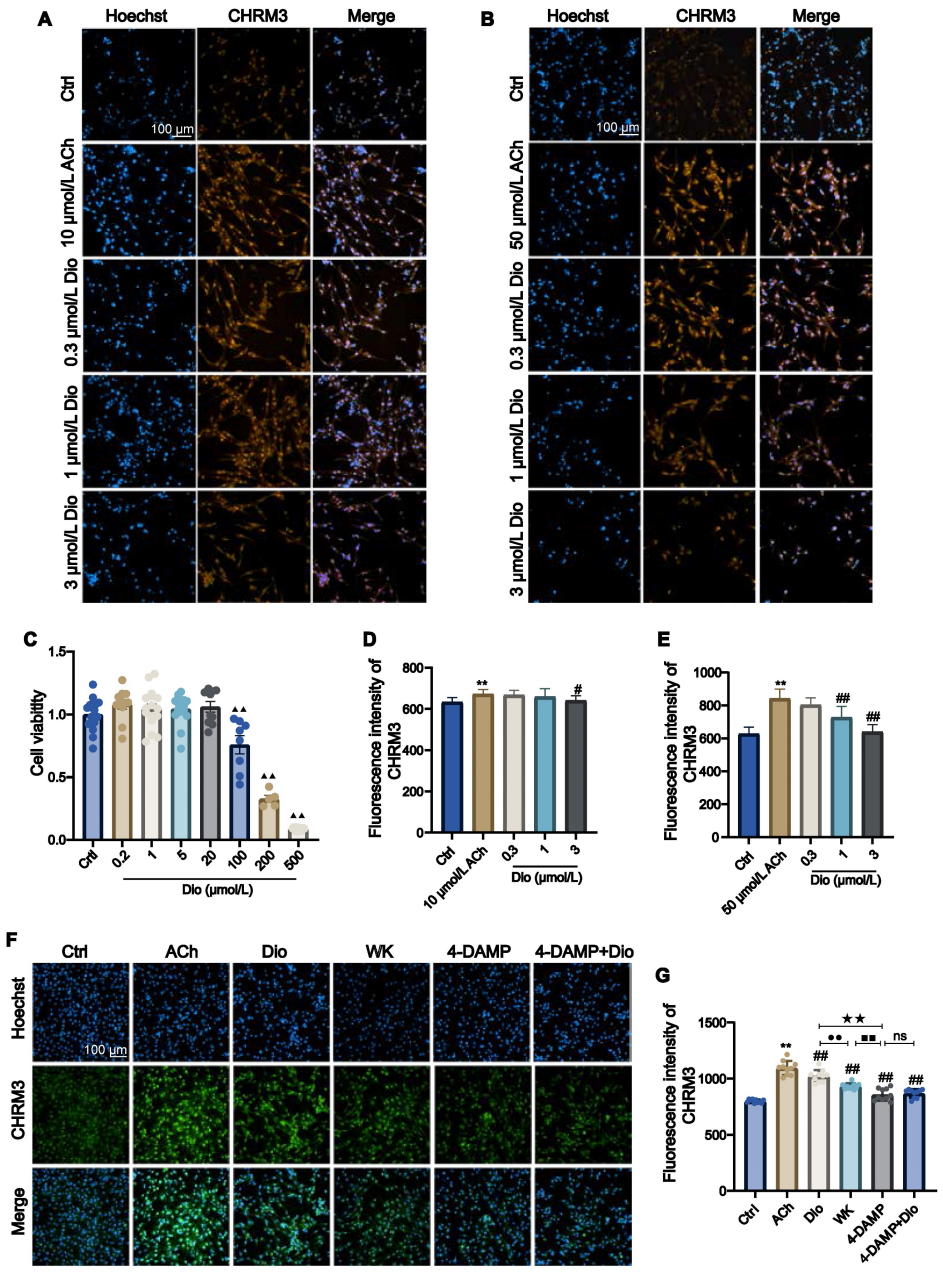
** The effect of WK active compounds on muscarinic acetylcholine receptor M3 (CHRM3) activation in HL-1 cells.** (A-E) Representative images and quantitation of HL-1 cells activated by 10 μmol/L ACh (A, D) or 50 μmol/L ACh (B, E) and blocked by 0.3-3 μmol/L Dioscin (Dio). The nuclei were stained in blue by Hoechst and the CHRM3 protein was stained in orange. Scale bar = 100 µm. (C) Bar graph quantitation of Dioscin on the viability of HL-1 cells. Values were expressed as mean ± SD (n = 4-5). (F-G) Representative images (F) and quantitation (G) of ACh-activated HL-1 cells and blocking effects by WK and Dioscin in the presence of 4-DAMP. The nucleus was stained blue, and CHRM3 were labeled green. Scale bar = 100 µm. Values were expressed as mean ± SD (n = 4-5). ^▲▲^P < 0.01 Dioscin vs. Ctrl,^ **^P < 0.01 vs ACh vs. Ctrl, ^#^P < 0.05 and ^##^P < 0.01 ACh vs. treat groups, ^●●^P < 0.01 Dioscin vs. WK, ^■■^P < 0.01 WK vs. 4-DAMP, ^★★^P < 0.01 Dioscin vs. 4-DAMP.

Since an inwardly rectifying K^+^ current is present in atrial cardiac myocytes that are activated by I_K-ACh_, atrial-derived cell line HL-1 was used to investigate the effect of Dioscin. CCK-8 assay determined that when the Dioscin concentration reached above 100 μmol/L (ranging from 0.2-500 μmol/L), the HL-1 cell viability was greatly decreased (**Figure 9C**). ACh (10 and 50 μmol/L) was employed to activate HL-1. **Figure 9D and E** showed that 3 μmol/L Dioscin reduced the CHRM3 expression compared to that in ACh group (3 μmol/L Dioscin = 641.22 ± 23.21 vs. 10 μmol/L ACh = 672.65 ± 21.67, 3 μmol/L Dioscin = 639.93 ± 43.87 vs. 50 μmol/L ACh = 842.27 ± 57.40, P < 0.01 or P < 0.05). As shown in **Figure 9E**, the CHRM3 expression in the 1 μmol/L Dioscin and 50 μmol/L ACh group was significantly reduced (1 μmol/L Dioscin = 728.42 ± 66.19 vs. 50 μmol/L Ach = 842.27 ± 57.40, P < 0.01). In addition, HL-1 cells treated with ACh showed clear activation of CHRM3 in contrast to the Ctrl (Ctrl = 799.00 ± 14.55 vs. ACh = 1094.70 ± 63.01, P < 0.01). The expression levels of CHRM3 in HL-1 cells generated by ACh were efficiently lowered by 1 mg/mL WK and 1 μmol/L Dioscin, which was consistent with the effects of 10 µmol/L 4-DAMP (an inhibitor of CHRM3, WK = 932.03 ± 26.18 vs. 4-DAMP = 857.14 ± 53.40, P < 0.01). Although there was no significant difference between 4-DAMP and 4-DAMP+Dioscin groups, there was a tendency of up-regulation (4-DAMP+Dioscin = 867.81 ± 37.81, P > 0.05, **Figure 9F and G**).

## Discussion

In this study, we established a rat model of ISIAF, characterized its electrophysiological properties, and evaluated the therapeutic efficacy of WK using a combination of optical mapping, whole-cell patch recording, RNA-seq, and network pharmacology analyses. Our results show that ischemic stroke-induced cardiac dysfunction includes AF, and this pathological process develops, at least in part, via a previously undocumented cholinergic synapse and calcium signaling mechanism in the atrium. The key findings of this study are: (1) Ischemic stroke led to significant cardiac Ca^2+^ handling heterogeneity, and a delay in the refractory period of CaT resulted in higher sensitivity to CaT and APD alternans, particularly spatially discordant alternans, which were causally linked to increased AF inducibility. These previously unreported cardiac phenotypes define ISIAF in a preclinical animal model; (2) WK alleviated ISIAF-related Ca^2+^ handling abnormalities and alternans; (3) ISIAF caused dramatic alterations in gene expression in the rat atrium, some of which were reversed by WK treatment. Notably, cholinergic synaptic signaling and calcium signaling pathways were the most affected in both ISIAF and by WK; (4) I_KM3_ and Ca_v1.2_ were the crucial ion channels involved in cholinergic synaptogenesis signaling and calcium signaling, respectively; (5) Dioscin, a key active component of WK, reduced M_3_ receptor expression and blocked the I_Ca-L_ current in the atria of ISIAF rats or activated HL-1 cells.

ISIAF is gaining increased clinical recognition. The finding that ischemic stroke frequently results in changes to the ECG was first reported in the 1950s and 1960s 53. Experimentally, although a shift in the cardiac calcium signal at 2 and 24 days following MCAO was observed in earlier studies 54,55, its relevance to brain injury-initiated cardiac calcium signaling dysfunction and arrhythmia was not explored. After a broad region of cerebral ischemia, investigations on the cardio-electrocardiogram and molecular characterization were conducted, but how specific electrical activity changes formed remained unclear. This lack of an experimental model for ISIAF has limited our ability to fully understand this syndrome and develop appropriate therapeutics for its treatment. To the best of our knowledge, this study was the first to use *ex vivo* optical mapping through Vm and Ca^2+^ signal channels to create a rat ISIAF model and investigate the precise atrial electrical pathways underlying its activation. In the ISIAF rats, gradual high-frequency stimulation of S1S1 with multiple AF/AT indicated poor tolerance of the atrium. The decrease in AERP/APD may increase the likelihood of reentry within the atria. In addition, the recovery period of the sinus node was prolonged, affecting its function. The ECG exhibited multiple episodes of AF/AT (Figure 1). Therefore, increased sensitivity and durability of AF, as well as greater susceptibility to rate-dependent atrial repolarization alternans, were verified in stroke-induced AF. At sinus rhythm and fixed frequency, the atrial APD and CaT were elevated in ISIAF rats, even prolonging the time to peak. Conduction velocity was reduced, and conduction direction disorder was amplified (Figure 2). Overall, the disturbance of electrical activity creates a vulnerable substrate for reentry at the whole heart level.

On the other hand, it is worth emphasizing the differences between our experimental model and the real clinical occurrence of ISIAF. For example, almost all animals that underwent the MCAO procedure in our Model group experienced atrial fibrillation, and the incidence/duration of arrhythmias was significantly different from those in the Sham group. This contrasts with clinical data, where only 25% of stroke patients develop AFDAS 12,13. One reason could be that, in the ISIAF model, the brain infarction was located in a specific region and maintained at a range of 26.21 ± 2.38%. Secondly, to assess the electrophysiological response, hearts were stimulated, while in real ischemic stroke patients, the area of cerebral infarction is not fixed, and the causes may be more complex. In our optical mapping study of cardiac tissue, 6 Hz stimulation was performed to adjust for rat heart rate, excluding the interference of sinus arrhythmia on conduction. We measured related indices of APD and Ca^2+^. Also, ERP was recorded when S1S2 was gradually shortened, and high-frequency stimulation was applied. Stable and gradual high-frequency stimulation of S1S1 was then performed again, and the electrical alternans and Ca^2+^ alternans were measured, along with the incidence and duration of arrhythmia. The susceptibility to arrhythmia in the isolated hearts of rats in each group was comprehensively assessed. Therefore, the true relevance of our model to the clinical ISIAF setting remains to be determined.

It has been reported that CaT alternans may serve as the principal driver of APD alternans 40,41. In the ISIAF atria, both APD and CaT alternans emerged at longer cycle lengths and their severity at shorter cycle durations was much greater than in the Sham atria. ISIAF rats were more susceptible to CaT alternans because disordered Ca^2^⁺ homeostasis, caused by malfunctioning RyR_2_ and SERCA, could not adapt to rapid pacing. CaT alternans in the model rats more readily developed spatial discordance when the cycle duration was extended to 100-50 ms. According to earlier research 56,57, the sensitivity to triggered and reentrant arrhythmias was exacerbated by repolarization heterogeneity when spatially discordant alternans occurred in the ISIAF atria. To understand the greater susceptibility of ISIAF atria to alternans, we investigated the refractory period of CaT, which strongly depends on the intrinsic refractoriness of RyR_2_ 57,58. In our study, ISIAF delayed RyR_2_ refractoriness and reduced the spatial dispersion of CaT recovery (Figure 3). This suggests that the alternans in APD, CaT, and spatial inconsistencies in ISIAF rats may be caused by delayed recovery of SR Ca^2+^ release and regional heterogeneity. Moreover, alternans in ISIAF rats could be linked to changes in APD caused by anomalies in repolarization ion channels (such as atria-specific I_K-ACh_ and I_Ca-L_) 24,41.

**Figure 10:**
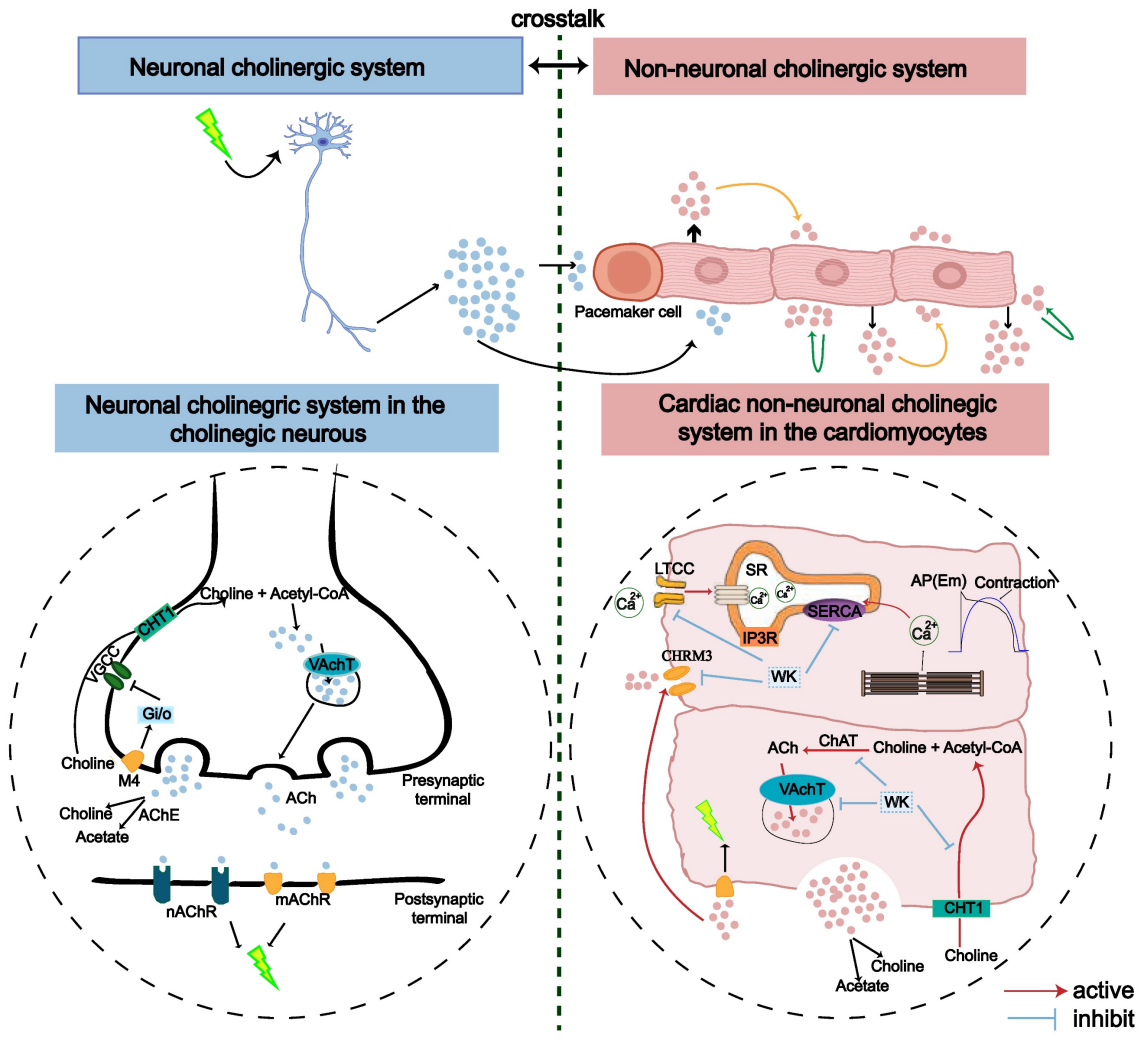
An illustration of the proposed role of cholinergic and calcium signaling pathways in ISIAF and WK treatment in neuronal and non-neuronal (cardiac) systems. The genes upregulated by ISIAF (CHAT, SLC5A7, AChE, CACNB1, ATP2A3, and CHRM3) were shown by the red arrow. Genes downregulated by WK (CHAT, SLC5A7, AChE, CACNB1, ATP2A3, and CHRM3) were marked in blue stripes.

It is generally considered that stroke-induced cardiac dysfunction is caused by autonomic and inflammatory mechanisms mediated by damage to the brain-heart axis connection 1. Particularly, the important roles of the sympathetic nerve, parasympathetic nerve, and hypothalamic-pituitary-adrenal (HPA) axis have been recognized 1,59-61. Moreover, a recent study by Yoshimoto *et al.* showed that different neurons project to specific brain regions to innervate parasympathetic neurons in the heart, controlling the heart rate 4. However, the molecular mechanism by which ischemic stroke induces AF remains unknown. Strikingly, our RNA-seq analysis revealed that cholinergic synaptic signaling was the most significantly altered pathway in the rat atrium after ischemic stroke (Figure 4, Sham vs. Model). Both neuronal and non-neuronal cholinergic system crosstalk mediate the physiological actions of the heart (Figure 10). Cardiomyocytes produce and release non-neuronal cholinergic signals by activating the non-neuronal cholinergic system (NNCS) 62, while acetylcholine is also known to play a pivotal role in neuroinflammation after stroke 1,7,61. It has been reported that the enzyme and receptor levels of acetylcholine change to varying degrees after cerebral ischemia-reperfusion injury. Acetylcholine exerts both presynaptic and postsynaptic effects through ionophilic nicotinic receptors (nAChRs) and mAChRs in the brain. While the role of nAChRs in stroke has received a lot of attention 63-65, the role of mAChRs is less understood. Our results showed that atrial M_3_ receptor gene expression significantly increased after ischemic stroke. Consistently, the enzymes and receptors regulating acetylcholine synthesis (ChAT), storage (VAChT), degradation (AChE), and reuptake for synthesis (CHT1) were also significantly upregulated in the atrial tissue of ISIAF rats (Figure 4) 21,64. Studies have shown that I_K-ACh_ is atrial-specific in the hearts of most animals, including humans. M_3_-mAChRs trigger delayed rectifying K^+^ currents (I_KM3_) to participate in cardiac repolarization, negative chronotropic effects, anti-dysrhythmic activity (suppressing ischemic dysrhythmias), and pro-dysrhythmic activity (accelerating AF) 66,67. It has been reported that spatial APD dispersion with alternations can cause AF in I_K-Ach_-activated cells 24. In support, our immunofluorescence data confirmed that CHRM3 was upregulated in ACh-activated HL-1 cells (Figure 9). Supporting this, our immunofluorescence data confirmed that CHRM3 was upregulated in ACh-activated HL-1 cells (Figure 9). In this context, it is interesting to note that a previous study showed that the cholinergic sympathetic pathway primes immunity in hypertension and mediates brain-to-spleen communication, providing a parallel case of the cholinergic sympathetic pathway mediating brain-to-organ communication 68.

Although stroke-induced AF has been clinically recognized and reported for decades, comprehensive preclinical studies have been lacking. Our finding that cholinergic synapse-calcium signaling mediates stroke-induced AF seems to diverge from the widely recognized role of the HPA axis, but it is supported by the following evidence: (1) Cholinergic synapse signaling was the most altered pathway in the ISIAF model atrium according to our RNA-seq results. However, other pathways may also be involved. This study has established a crosstalk between cholinergic synapse signaling and calcium signaling pathways (Figure 10). (2) The cerebral hemisphere implicated in a stroke determines the impact on the cardiovascular system, a process known as lateralization 69,70. However, it has been suggested that the clinical relevance of cardiovascular autonomic function lateralization remains uncertain 71. (3) Traditional electrophysiological assays are primarily used to evaluate single cells and rely on individualized methods like patch clamping and ECG. However, these approaches lack intuitive electrophysiological markers at the tissue level. We employed the optical mapping technique to compare different electrophysiological indices and obtain standardized differences between groups with the same stimulation frequency. This allows for a more intuitive and accurate evaluation of the susceptibility and mechanisms of atrial arrhythmia.

The anti-arrhythmic properties of WK are well-documented 72. Our pharmacological and virtual screenings of WK compounds have identified potential anti-arrhythmic molecules, including neurotransmitters 73. We recently demonstrated that WK possesses antiplatelet activity which helps lower the risk of stroke recurrence 74. However, whether WK can treat AF caused by ischemic stroke remained unknown until this study. We demonstrate WK's efficacy against ISIAF across multiple levels: overall ECG, tissue, cell layers, and single cells. WK treatment improves parameters such as Toff and Tau, suggesting its effects are related to improved SERCA function. It not only reduced the dispersion of CaT but also shortened the prolonged time to peak and CaT in ISIAF atria, implying that WK therapy normalizes calcium handling by addressing abnormal SR calcium release and absorption throughout the atria. Additionally, WK reduced alternans in APD, CaT, and spatial inconsistencies by reversing the delay and dispersion in RyR refractoriness (Figures 2-3). In ISIAF atria, WK shifted the I_Ca-L_ peak current and I-V curves upward. Since the I-V curve shape, peak current activation potential, reversal potential, and threshold potential remained unchanged. It may not affect the rectification properties or ion selectivity of I_Ca-L_ channels. However, WK extended the activation window and decreased the V_1/2_ of the activation curve (Figure 5), suggesting that WK inhibits I_Ca-L_ by impacting the time-dependent activation and voltage-dependent deactivation of I_Ca-L_ channels 51,75. Mechanistically, our RNA-seq analysis revealed that the cholinergic synaptic signaling genes most significantly altered in the rat atrium after ischemic stroke were also prominently reversed by WK (Figure 4, Model vs. WK), suggesting that WK may contain active ingredients capable of regulating the cholinergic synaptic-calcium axis. Interestingly, in a previous study on stroke-heart syndrome, we found that a different Chinese medicine, Danhong injection, protected against ischemic stroke-induced cardiac dysfunction via the HPA-mediated adrenergic pathway *in vivo* or AVP signaling* in vitro* 76,77. That earlier study focused on ventricular dysfunction and sympathetic nervous system involvement, emphasizing the HPA axis's role. However, our current RNA-seq data demonstrate that while adrenergic signaling in cardiomyocytes was affected, parasympathetic nerve and cholinergic synapse-calcium signaling were more critical in AF development. The diverse model-dependent cardiac abnormalities and the distinct therapeutic effects of different compound herbal medicines merit further exploration in future investigations.

We employed molecular docking and whole-cell patch-clamp screening techniques to identify the potential active components of WK that interact with cholinergic receptors and downstream I_Ca-L_/I_KM3_ ion channels. Among the top candidates, we discovered that while Tanshinone-IIA exhibited a high binding affinity for CACNB1 and CHRM3, it did not significantly influence I_Ca-L_. In contrast, Dioscin, which ranked second in binding to CACNB1 and CHRM3, effectively reduced I_Ca-L_ (Figures 7-8). Additionally, Dioscin downregulated the expression of CHRM3 in HL-1 atrial cells (Figure 9). This finding is consistent with previous reports showing that Dioscin protects the heart in various conditions, such as arrhythmia, ischemia/reperfusion injury, hypertrophy, and doxorubicin-induced cardiotoxicity 78, improves cardiac function by upregulating antioxidants 79,80, preserves mitochondrial function, prevents remodeling in acute myocardial infarction 81, inhibits migration and angiogenesis in hypoxic cells, and alleviates hypertrophy 82,83. Our results suggest a new therapeutic target for Dioscin in treating cardiac dysfunction, specifically through the inhibition of I_Ca-L_ and I_KM3_, which reduces susceptibility to ISIAF.

## Conclusions

Our study is the first to establish a rat model of ISIAF and systematically characterize the atrial electrophysiological changes following cerebral ischemia. Molecular mechanistic analyses revealed that the cholinergic synapse and calcium signaling pathways play a central role in this brain-heart syndrome. Notably, ISIAF is partially reversible through treatment with WK, a compound Chinese medicine, which exerts its effects by regulating the same cholinergic synapse-calcium signaling pathway. One of its active components, Dioscin, acts directly by binding to I_KM3_ and inhibiting I_Ca-L_.

### Limitations

When evaluating our findings, a few limitations need to be considered. First, the optical mapping study presented here used the isolated and denervated heart. Since the events we examined may entail nerve injury, corresponding *in vivo* experiments with nerve stimulation are required in the future to supplement the *ex vivo* data. Second, it is well known that Ca^2+^ handling anomalies at the cellular level, which are caused by changes in Ca^2+^ handling proteins and result in proarrhythmic DADs, act as a catalyst for atrial arrhythmia. DADs may also exist in atrial cardiomyocytes derived from model group rats; these cells should be further investigated utilizing Ca^2+^ imaging. Third, Met is a medicine that is generally effective, and it is well known that Met is frequently used as a β-blocker. The effect of Met was obvious, demonstrating the role of the β-receptor in atrial fibrillation. Fourth, look at systems where ACh receptor function is not the source of calcium signaling pathways. Because MCAO has a very broad wound area, its process is complicated and includes both systemic and local inflammation in addition to several other neurotransmitters and second messengers.

This study is about the mechanisms that cause tremors in a stroke, but it is not known whether WK works in the area of cerebral and heart cooperation, which will be one of the points we will study in the future. Meanwhile, we believe that there is a certain limitation to RNA-seq and that the entire choline-energy signal pathway is not fully matched. However, through our RT-PCR validation and IF experiments on CHRM3 receptors, we can determine the role of the CHRM3 receptor in the signal transduction process.

## Supplementary Material

Supplementary figures, method and table.

Supplementary RNA-seq differential expression data.

## Figures and Tables

**Table 1:** Main chemical compounds in aqueous solution or WK-treated rat plasma.

No.	tR/min	Formula	Ion mode	Measured mass	Calculated mass	Error (ppm)	MS/MS fragmentation	Name	Herb
1	1.23	C6H12O6	[M-H]^-^	179.0558	179.0556	-1.1	179.0558	GUP *	1,2,3
2	3.13	C14H21NO4	[M+H]^+^	268.1546	268.1549	1.1	268.1546	Codonopsine	1
3	4.84	C7H8O3	[M+Na]^+^	163.0385	163.0371	-8.6	163.0395,141.0588	5-Methoxymethyl furfura	1
4	5.67	C19H20O3	[M-H]^-^	295.1326	295.1334	2.7	295.1326	Cryptotanshinon*	3
5	5.68	C19H18O3	[M-H]^-^	293.1163	293.1178	5.1	293.1163	Tanshinone IIA *	3
6	6.56	C18H34O11	[M-H]^-^	425.2039	425.2023	-3.8	471.2065,425.2039	Hexyl-β-D-glucopyranosyl-(1→2)-β-D-glucopyranoside*	1
7	7.99	C11H12O3	[M+H]^+^	193.0871	193.0865	-3.1	193.0871	Myristicin	1
8	8.26	C9H16O4	[M-H]^-^	187.0972	187.0970	-1.1	187.0972, 125.8719	Azelaic acid*	1
9	8.37	C20H28O8	[M+Na]^+^	419.1686	419.1682	-1.0	419.1686	Lobetyolin	1
10	8.41	C39H60O14	[M+H]^+^	753.4073	753.4061	-1.6	753.4071	Kingianoside A*	4
11	8.42	C47H80O18	[M-H]^-^	931.5261	931.5266	0.5	977.5416,931.5261	Notoginsenoside R1*	1,2
12	8.97	C36H62O8	[M+H]^+^	623.4547	623.4523	-3.8	623.4547	Ginsenoside Rh2	2
13	8.98	C15H22	[M+H]^+^	203.1789	203.1800	5.4	203.1789	a-Curcumene	1
14	9.00	C42H72O14	[M-H]^-^	799.4868	799.4844	-3.0	845.4943,799.4868,637.4395	Ginsenoside Rf*	2
15	9.01	C42H72O14	[M-H]^-^	799.4868	799.4844	-3.0	845.5002,799.4868,637.4344	Ginsenoside Rg1*	2
16	9.02	C48H82O18	[M-H]^-^	945.5404	945.5423	2.0	991.5484,945.5404,799.4868	Ginsenoside Re*	2
17	10.41	C15H20O2	[M-H]^-^	231.1571	231.1585	6.1	276.0876,231.1571	Atractylenolide II*	1
18	10.41	C17H26O3	[M+H]^+^	279.1946	279.1960	5.0	279.1946	Panaxytrio	2
19	12.77	C64H108O31	[M-H]^-^	1371.6796	1371.6871	5.5	1417.6914,1371.6796	Notoginsenoside T	2
20	13.47	C59H100O27	[M-H]^-^	1239.6395	1239.6374	-1.7	1285.6453,1239.6395	Notoginsenoside Fa*	2
21	13.84	C41H70O13	[M-H]^-^	769.4752	769.4738	-1.8	769.4752,637.4292,475.3807	Notoginsenoside R2	2
22	14.09	C59H100O27	[M-H]^-^	1240.6395	1240.6374	-1.7	1285.6453	Notoginsenoside R4*	2
23	14.34	C30H42O8	[M+COOH]^-^	575.2887	575.2856	-5.4	1151.5875,575.2887	Dehydroadynerigenin digitaloside	4
24	14.73	C42H72O13	[M-H]^-^	783.3871	783.3895	3.1	783.3871	Ginsenoside Rg3	2
25	14.73	C36H62O9	[M-H]^-^	683.4370	683.4418	7.0	683.4370	Ginsenoside Rh1	2
26	14.87	C54H92O23	[M-H]^-^	1107.5985	1107.5951	-3.1	1153.6105,1107.5685,783.4832	Ginsenoside Rb1*	2
27	15.24	C36H62O9	[M+COOH]^-^	683.4370	683.4364	-0.9	683.4370	Sanchinoside B1	2
28	15.26	C36H60O8	[M+H]^+^	621.4378	621.4366	-1.9	1195.6073,621.4378	Ginsenoside Rh4	2
29	15.47	C58H98O26	[M-H]^-^	1209.6293	1209.6268	-2.1	1209.6293	Notoginsenoside Fc	2
30	16.10	C53H90O22	[M-H]^-^	1079.5947	1079.6002	5.1	1123.5958,1079.5947	Ginsenoside Rb2*	2
31	16.11	C53H90O22	[M-H]^-^	1077.5807	1077.5845	3.5	1123.5890,1077.5807,620.3041	Ginsenoside Rb3*	2
32	16.31	C53H90O22	[M-H]^-^	1077.5874	1077.5845	-2.7	1123.5890,1077.5874,785.4677	Ginsenoside Rc*	2
33	17.46	C48H82O18	[M-H]^-^	945.5404	945.5423	2.0	991.5549,945.5404,783.4832	Ginsenoside Rd*	2
34	18.27	C15H22O3	[M-H]^-^	249.1481	249.1491	4.0	249.1481	Nardosinone*	3
35	19.19	C45H72O16	[M+COOH]^-^	913.4804	913.4797	-0.8	913.4804	Dioscin*	4
36	19.81	C47H80O17	[M-H]^-^	915.5272	915.5317	4.9	961.5316, 915.5272	Notoginsenoside Fe	2
37	19.82	C48H82O19	[M-H]^-^	961.5380	961.5372	-0.8	1033.5433,961.5380	Notoginsenoside R3	2
38	20.26	C48H82O19	[M-H]^-^	961.5316	961.5372	5.8	1033.5477,961.5316	Notoginsenoside R6	2
39	24.03	C42H72O13	[M-H]^-^	783.4832	783.4895	8.0	829.4944,783.4832,	Ginsenoside Rg2	2
40	28.29	C16H22O4	[M-H]^-^	277.1433	277.1440	2.5	277.1433	Ditertbutyl phthalate*	2

*The rat plasma obtained after 1h WK treatment. 1: Codonopsis pilosula (Franch.) Nannf.; 2: Panax notoginseng (Burkill) F.H.Chen; 3: Nardostachys jatamansi (D.Don) DC.; 4: Polygonatum kingianum Collett & Hemsl.

## Abbreviations

ACh: acetylcholine
AERP: atrial effective refractive period
AF: atrial fibrillation
AFDAS: AF detected after stroke
AT: atrial tachycardia
ISIAF: ischemic stroke-induced AF
AIS: acute ischemic stroke
ANS: autonomic nervous system
APD: action potential duration
CaT: Ca^2+^ transient duration
CACNB1: calcium voltage-gated channel auxiliary subunit beta 1
CCA: common carotid artery
CHRM3: muscarinic acetylcholine receptor M3
DADs: delayed after depolarizations
DEGs: differentially expressed genes
Dio: Dioscin
ECA: external cerebral artery
GPs: ganglionated plexi
GO: Gene Ontology
ICA: internal cerebral artery
I_KM3_: M3 muscarinic acetylcholine receptors trigger delayed rectifying K^+^ currents
IQR: interquartile interval
HPA: hypothalamic-pituitary-adrenal
ICNS: intrinsic cardiac nervous system
I_K-ACh_: ACh-activated potassium current
KEGG: Kyoto Encyclopedia of Genes and Genomes
LTCCs: L-type calcium channels
mAChR: muscarinic ACh receptors
MCAO: middle cerebral artery occlusion
Met: Metoprolol
nAChR: ionophilic nicotinic receptors
RyR_2_: ryanodine receptor 2
SERCA: sarcoplasmic reticulum Ca^2+^-ATPase
SHS: Stroke heart syndrome
SIHI: stroke induced heart injury
SR: sarcoplasmic reticulum
SNS: sympathetic nervous systems
Tau: intracellular Ca^2+^ decay
TCM: traditional Chinese medicine
TTC: 2,3,5-Triphenyltetrazolium chloride
WK: Wenxin Keli

## References

[1] Sposato LA, Hilz MJ, Aspberg S, Murthy SB, Bahit MC, Hsieh CY (2020). Post-stroke cardiovascular complications and neurogenic cardiac injury. J Am Coll Cardiol.

[2] Liu W, Zhang X, Wu Z, Huang K, Yang C, Yang L (2022). Brain-heart communication in health and diseases. Brain Res Bull.

[3] Doehner W, Böhm M, Boriani G, Christersson C, Coats AJS, Haeusler KG (2023). Interaction of heart failure and stroke: a clinical consensus statement of the ESC council on stroke, the heart failure association (HFA) and the ESC working group on thrombosis. Eur J Heart Fail.

[4] Yoshimoto A, Morikawa S, Kato E, Takeuchi H, Ikegaya Y (2024). Top-down brain circuits for operant bradycardia. Science.

[5] Méloux A, Béjot Y, Rochette L, Cottin Y, Vergely C (2020). Brain-heart interactions during ischemic processes: clinical and experimental evidences. Stroke.

[6] Sposato LA, Lam M, Allen B, Richard L, Shariff SZ, Saposnik G (2020). First-ever ischemic stroke and increased risk of incident heart disease in older adults. Neurology.

[7] Scheitz JF, Nolte CH, Doehner W, Hachinski V, Endres M (2018). Stroke-heart syndrome: clinical presentation and underlying mechanisms. Lancet Neurol.

[8] Balint B, Jaremek V, Thorburn V, Whitehead SN, Sposato LA (2019). Left atrial microvascular endothelial dysfunction, myocardial inflammation and fibrosis after selective insular cortex ischemic stroke. Int J Cardiol.

[9] Sposato LA, Fridman S, Whitehead SN, Lopes RD (2018). Linking stroke-induced heart injury and neurogenic atrial fibrillation: a hypothesis to be proven. J Electrocardiol.

[10] Benjamin EJ, Muntner P, Alonso A, Bittencourt M, Callaway CW, Carson AP (2019). Heart disease and stroke statistics-2019 update: a report from the American heart association. Circulation.

[11] Liu Q, Zhao J, Wang S (2022). From cerebrovascular diseases to neuro-co-cardiological diseases: a challenge in the new epoch. Sci Bull.

[12] Bernstein RA, Kamel H, Granger CB, Piccini JP, Sethi PP, Katz JM (2021). Effect of long-term continuous cardiac monitoring vs usual care on detection of atrial fibrillation in patients with stroke attributed to large- or small-vessel disease. JAMA.

[13] Buckley BJR, Harrison SL, Hill A, Underhill P, Lane DA, Lip GYH (2022). Stroke-heart syndrome: incidence and clinical outcomes of cardiac complications following stroke. Stroke.

[14] Cerasuolo JO, Cipriano LE, Sposato LA (2017). The complexity of atrial fibrillation newly diagnosed after ischemic stroke and transient ischemic attack: advances and uncertainties. Curr Opin Neurol.

[15] Sposato LA, Riccio PM, Hachinski V (2014). Poststroke atrial fibrillation: cause or consequence? critical review of current views. Neurology.

[16] Scheitz JF, Sposato LA, Schulz-Menger J, Nolte CH, Backs J, Endres M (2022). Stroke-heart syndrome: recent advances and challenges. JAHA.

[17] Wang L, Olivas A, Francis Stuart SD, Tapa S, Blake MR, Woodward WR (2020). Cardiac sympathetic nerve transdifferentiation reduces action potential heterogeneity after myocardial infarction. Am J Physiol Heart Circ Physiol.

[18] Armour JA, Murphy DA, Yuan B, MacDonald S, Hopkins DA (1997). Gross and microscopic anatomy of the human intrinsic cardiac nervous system. Anat Rec.

[19] Singh S, Johnson PI, Lee RE, Orfei E, Lonchyna VA, Sullivan HJ (1996). Topography of cardiac ganglia in the adult human heart. J Thorac Cardiovasc Surg.

[20] Rysevaite K, Saburkina I, Pauziene N, Noujaim S, Jalife J, Pauza DH (2011). Morphologic pattern of the intrinsic ganglionated nerve plexus in the mouse heart. Heart Rhythm.

[21] Parsons SM, Prior C, Marshall IG (1993). Acetylcholine transport, storage, and release. Int Rev Neurobiol.

[22] Vaseghi M, Barwad P, Malavassi Corrales FJ, Tandri H, Mathuria N, Shah R (2017). Cardiac sympathetic denervation for refractory ventricular arrhythmias. J Am Coll Cardiol.

[23] Roy A, Fields WC, Rocha-Resende C, Resende RR, Guatimosim S, Prado VF (2013). Cardiomyocyte-secreted acetylcholine is required for maintenance of homeostasis in the heart. FASEB J.

[24] Bingen BO, Neshati Z, Askar SFA, Kazbanov IV, Ypey DL, Panfilov AV (2013). Atrium-specific Kir3.x determines inducibility, dynamics, and termination of fibrillation by regulating restitution-driven Alternans. Circulation.

[25] Wang Z, Shi H, Wang H (2004). Functional M_3_ muscarinic acetylcholine receptors in mammalian hearts: characterization of cardiac M_3_ receptors. Br J Pharmacol.

[26] Dobrev D, Nattel S (2008). Calcium handling abnormalities in atrial fibrillation as a target for innovative therapeutics. J Cardiovasc Pharmacol.

[27] Dobrev D, Wehrens XHT (2017). Calcium-mediated cellular triggered activity in atrial fibrillation. J Physiol.

[28] Fakuade FE, Steckmeister V, Seibertz F, Gronwald J, Kestel S, Menzel J (2021). Altered atrial cytosolic calcium handling contributes to the development of postoperative atrial fibrillation. Cardiovasc Res.

[29] Venetucci LA, Trafford AW, Eisner DA (2007). Increasing ryanodine receptor open probability alone does not produce arrhythmogenic calcium waves: threshold sarcoplasmic reticulum calcium content is required. Circ Res.

[30] Santiago DJ, Ríos E, Shannon TR (2013). Isoproterenol increases the fraction of spark-dependent RyR-mediated leak in ventricular myocytes. Biophys J.

[31] Hofmann F, Flockerzi V, Kahl S, Wegener JW (2014). L-type Ca_V1.2_ calcium channels: from in vitro findings to in vivo function. Physiol Rev.

[32] Shaw RM, Colecraft HM (2013). L-type calcium channel targeting and local signalling in cardiac myocytes. Cardiovasc Res.

[33] Hao P, Jiang F, Cheng J, Ma L, Zhang Y, Zhao Y (2017). Traditional Chinese medicine for cardiovascular disease. J Am Coll Cardiol.

[34] Wang X, Wang X, Gu Y, Wang T, Huang C (2013). Wenxin keli attenuates ischemia-induced ventricular arrhythmias in rats: involvement of L-type calcium and transient outward potassium currents. Mol Med Rep.

[35] Luo A, Liu Z, Cao Z, Hao J, Wu L, Fu C (2017). Wenxin keli diminishes Ca^2+^ overload induced by hypoxia/reoxygenation in cardiomyocytes through inhibiting I_NaL_ and I_CaL_. Pacing Clin Electrophysiol.

[36] Chen Y, Li Y, Guo L, Chen W, Zhao M, Gao Y (2013). Effects of wenxin keli on the action potential and L-type calcium current in rats with transverse aortic constriction-induced heart failure. Evid Based Complement Alternat Med.

[37] Liu K, Lv M, Ji X, Lou L, Nie B, Zhao J (2021). Wenxin granules regulate endoplasmic reticulum stress unfolded protein response and improve ventricular remodeling on rats with myocardial infarction. Evid Based Complement Alternat Med.

[38] Wang Y, He S, Liu X, Li Z, Zhu L, Xiao G (2021). Galectin-3 mediated inflammatory response contributes to neurological recovery by qishenyiqi in subacute stroke model. Front Pharmacol.

[39] Fedorov VV, Lozinsky IT, Sosunov EA, Anyukhovsky EP, Rosen MR, Balke CW (2007). Application of blebbistatin as an excitation-contraction uncoupler for electrophysiologic study of rat and rabbit hearts. Heart Rhythm.

[40] Wang L, Myles RC, Jesus NMD, Ohlendorf AKP, Bers DM, Ripplinger CM (2014). Optical mapping of sarcoplasmic reticulum Ca^2+^ in the intact heart: ryanodine receptor refractoriness during alternans and fibrillation. Circ Res.

[41] Liu T, Xiong F, Qi X, Xiao J, Villeneuve L, Taha IA (2020). Altered calcium handling produces reentry-promoting action potential alternans in atrial fibrillation-remodeled hearts. JCI Insight.

[42] Liao J, Zhang S, Yang S, Lu Y, Lu K, Wu Y (2021). Interleukin-6-Mediated-Ca^2+^ handling abnormalities contributes to atrial fibrillation in sterile pericarditis rats. Front Immunol.

[43] Xie D, Xiong K, Su X, Wang G, Ji Q, Zou Q (2021). Identification of an endogenous glutamatergic transmitter system controlling excitability and conductivity of atrial cardiomyocytes. Cell Res.

[44] Siedlecka U, Arora M, Kolettis T, Soppa GKR, Lee J, Stagg MA (2008). Effects of clenbuterol on contractility and Ca^2+^ homeostasis of isolated rat ventricular myocytes. Am J Physiol Heart Circ Physiol.

[45] Linnenbank AC, De Bakker JMT, Coronel R (2014). How to measure propagation velocity in cardiac tissue: a simulation study. Front Physiol.

[46] Heijman J, Muna AP, Veleva T, Molina CE, Sutanto H, Tekook M (2020). Atrial myocyte NLRP3/CaMKII nexus forms a substrate for postoperative atrial fibrillation. Circ Res.

[47] Voigt N, Heijman J, Wang Q, Chiang DY, Li N, Karck M (2014). Cellular and molecular mechanisms of atrial arrhythmogenesis in patients with paroxysmal atrial fibrillation. Circulation.

[48] Naumenko N, Mutikainen M, Holappa L, Ruas JL, Tuomainen T, Tavi P (2022). PGC-1α deficiency reveals sex-specific links between cardiac energy metabolism and EC-coupling during development of heart failure in mice. Cardiovasc Res.

[49] Psaras Y, Margara F, Cicconet M, Sparrow AJ, Repetti GG, Schmid M (2021). CalTrack: high-throughput automated calcium transient analysis in cardiomyocytes. Circ Res.

[50] O'Shea C, Holmes AP, Yu TY, Winter J, Wells SP, Correia J (2019). ElectroMap: high-throughput open-source software for analysis and mapping of cardiac electrophysiology. Sci Rep.

[51] Liu Z, Hu L, Zhang Z, Song L, Zhang P, Cao Z (2021). Isoliensinine eliminates afterdepolarizations through inhibiting late sodium current and L-type calcium current. Cardiovasc Toxicol.

[52] Sun L, Du J, Zhang G, Zhang Y, Pan G, Wang L (2008). Aberration of L-type calcium channel in cardiac myocytes is one of the mechanisms of arrhythmia induced by cerebral ischemia. Cell Physiol Biochem.

[53] Burch GE, Meyers R, Abildskov JA (1954). A new electrocardiographic pattern observed in cerebrovascular accidents. Circulation.

[54] Wang L, Sun L, Zhang Y, Wu H, Li C, Pan Z (2009). Ionic mechanisms underlying action potential prolongation by focal cerebral ischemia in rat ventricular myocytes. Cell Physiol Biochem.

[55] Bers DM (2002). Cardiac excitation-contraction coupling. Nature.

[56] Hayashi H, Shiferaw Y, Sato D, Nihei M, Lin SF, Chen PS (2007). Dynamic origin of spatially discordant alternans in cardiac tissue. Biophys J.

[57] Schotten U, Verheule S, Kirchhof P, Goette A (2011). Pathophysiological mechanisms of atrial fibrillation: a translational appraisal. Physiol Rev.

[58] Greiser M, Lederer WJ, Schotten U (2011). Alterations of atrial Ca^2+^ handling as cause and consequence of atrial fibrillation. Cardiovasc Res.

[59] Chen Z, Venkat P, Seyfried D, Chopp M, Yan T, Chen J (2017). Brain-heart interaction: cardiac complications after stroke. Circulation research.

[60] Battaglini D, Robba C, Silva ALD, Samary CDS, Silva PL, Pizzol FD (2020). Brain-heart interaction after acute ischemic stroke. Crit Care.

[61] Samuels MA (2007). The brain-heart connection. Circulation.

[62] Ardell JL, Rajendran PS, Nier HA, KenKnight BH, Armour JA (2015). Central-peripheral neural network interactions evoked by vagus nerve stimulation: functional consequences on control of cardiac function. Am J Physiol Heart Circ Physiol.

[63] Benarroch EE (2010). Acetylcholine in the cerebral cortex: effects and clinical implications. Neurology.

[64] Picciotto MR, Higley MJ, Mineur YS (2012). Acetylcholine as a neuromodulator: cholinergic signaling shapes nervous system function and behavior. Neuron.

[65] Ferreira-Vieira TH, M Guimaraes I, R Silva F, M Ribeiro F (2016). Alzheimer's disease: targeting the cholinergic system. CN.

[66] Liu Y, Xu C, Jiao J, Wang H, Dong D, Yang B (2005). M_3_-R/I_KM3_-a new target of antiarrhythmic agents. Yao Xue Xue Bao.

[67] Benavides-Haro DE, Navarro-Polanco RA, Sánchez-Chapula JA (2003). The cholinomimetic agent bethanechol activates I_K-ACh_ in feline atrial myocytes. Naunyn Schmiedebergs Arch Pharmacol.

[68] Perrotta M (2016). A cholinergic-sympathetic pathway primes immunity in hypertension and mediates brain-to-spleen communication. Nat Commun.

[69] Hilz MJ, Dütsch M, Perrine K, Nelson PK, Rauhut U, Devinsky O (2001). Hemispheric influence on autonomic modulation and baroreflex sensitivity. Ann Neurol.

[70] Oppenheimer SM, Gelb A, Girvin JP, Hachinski VC (1992). Cardiovascular effects of human insular cortex stimulation. Neurology.

[71] Jaremek VM, Whitehead S, Sposato LA (2019). Lateralization of the control of cardiovascular autonomic function and left atrial injury after selective right and left insular stroke. Int J Cardiol.

[72] Burashnikov A, Petroski A, Hu D, Martinez HB, Antzelevitch C (2012). Atrial-selective inhibition of sodium-channel current by Wenxin Keli is effective in suppressing atrial fibrillation. Heart Rhythm.

[73] Wang T, Lu M, Du Q, Yao X, Zhang P, Chen X (2017). An integrated anti-arrhythmic target network of a Chinese medicine compound, wenxin keli, revealed by combined machine learning and molecular pathway analysis. Mol BioSyst.

[74] Tao Q, Xiao G, Wang T, Zhang L, Yu M, Peng L (2022). Identification of linoleic acid as an antithrombotic component of wenxin keli via selective inhibition of p-selectin-mediated platelet activation. Biomed Pharmacother.

[75] Liang Y, Zhang Y, Liu M, Han X, Zhang J, Zhang X (2020). Protective effect of quercetin against myocardial ischemia as a Ca^2+^ channel inhibitor: involvement of inhibiting contractility and Ca^2+^ influx via L-type Ca^2+^ channels. Arch Pharm Res.

[76] Yang M, Orgah J, Zhu J, Fan G, Han J, Wang X (2016). Danhong injection attenuates cardiac injury induced by ischemic and reperfused neuronal cells through regulating arginine vasopressin expression and secretion. Brain Res.

[77] Orgah J, Yu J, Zhao T, Wang L, Yang M, Zhang Y (2018). Danhong injection reversed cardiac abnormality in brain-heart syndrome via local and remote β-adrenergic receptor signaling. Front Pharmacol.

[78] Li X, Liu S, Qu L, Chen Y, Yuan C, Qin A (2021). Dioscin and diosgenin: insights into their potential protective effects in cardiac diseases. J Ethnopharmacol.

[79] Cheng J, Sun C, Zhang J, Zou Q, Hao Q, Xue Y (2019). The protective effects of preconditioning with Dioscin on myocardial ischemia/reperfusion-induced ventricular arrhythmias by increasing connexin 43 expression in rats. J Cardiovasc Pharmacol Ther.

[80] Lyu D, Tian Q, Qian H, He C, Shen T, Xi J (2021). Dioscin attenuates myocardial ischemic/reperfusion-induced cardiac dysfunction through suppression of reactive oxygen species. Oxid Med Cell Longev.

[81] Shen T, Lyu D, Zhang M, Shang H, Lu Q (2022). Dioscin Alleviates cardiac dysfunction in acute myocardial infarction via rescuing mitochondrial malfunction. Front Cardiovasc Med.

[82] Kong C, Lyu D, He C, Li R, Lu Q (2021). Dioscin elevates lncRNA MANTIS in therapeutic angiogenesis for heart diseases. Aging Cell.

[83] Yang B, Xu B, Zhao H, Wang Y, Zhang J, Li C (2018). Dioscin protects against coronary heart disease by reducing oxidative stress and inflammation via Sirt1/Nrf2 and p38 MAPK pathways. Mol Med Rep.

