# Supplementary material for: Brain-to-heart cholinergic synapse-calcium signaling mediates ischemic stroke-induced atrial fibrillation
Source: Theranostics. 2024 Oct 7;14(17):6625–51. doi: 10.7150/thno.99065 (PMC11519791; doi:10.7150/thno.99065)
Supplement: Supplementary file 1 — Supplementary figures, method and table. [file thnov14p6625s1.pdf]

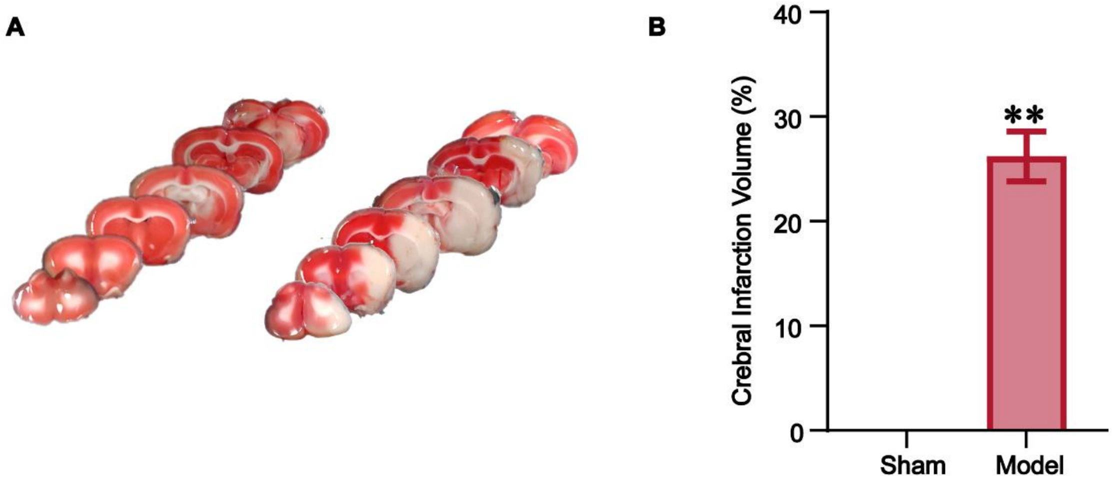

Figure S1. Gold standard TTC for identifying MCAO model success. (A) Representative pictures of each group's TTC-stained brain slices. The normal portions were red, whereas the infarct areas were shown in white. (B) A quantitative examination of each group's cerebral infarction volumes (n = 8). Values were expressed as mean ± SD (n = 5). $^{*}P < 0.05$ and $^{**}P < 0.01$ vs. Sham group, $^{\#\#}P < 0.01$ vs. Model group.

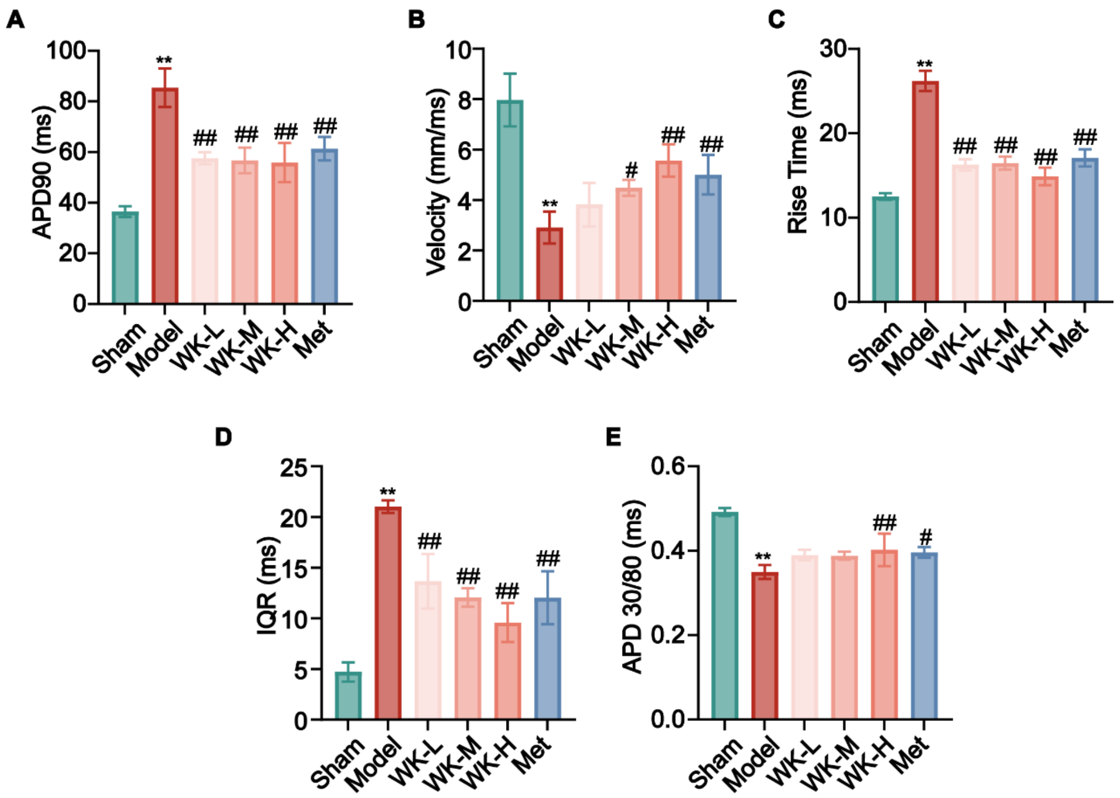

Figure S2. Each group involves in the APD via ex vivo optical mapping. (A) Quantification of APD90 in six groups. (B) Calculation of velocity in AP phase each right atrium of six groups. (C) Calculation of rise time in AP phase each right atrium of six groups. (D) Calculation of IQR in AP phase each right atrium of six groups. (E) Statistical results of APD 30/80 in six groups. Values were expressed as mean ± SD ($n = 5$). $^{*}P < 0.05$ and $^{**}P < 0.01$ vs. Sham group, $^{\#}P < 0.05$ and $^{\#\#}P < 0.01$ vs. Model group.

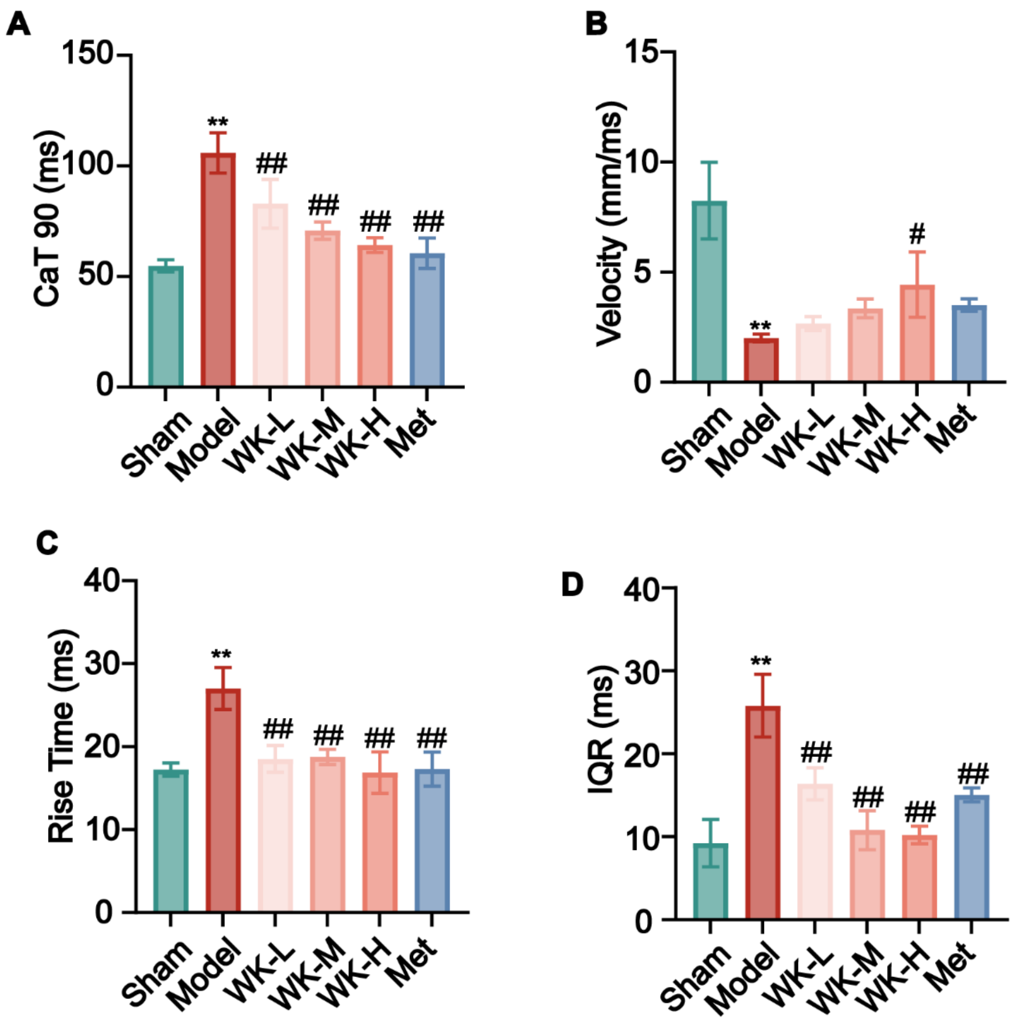

Figure S3. Each group involves in the CaT alterations via ex vivo optical mapping. (A) Quantification of CaT90 in six groups. (B) Calculation of velocity in Ca release each right atrium of six groups. (C) Calculation of rise time in Ca release each right atrium of six groups. (D) Calculation of IQR in Ca release each right atrium of six groups. Values were expressed as mean ± SD (n = 5). $^{*}P < 0.05$ and $^{**}P < 0.01$ vs. Sham group, $^{\#}P < 0.05$ and $^{\#\#}P < 0.01$ vs. Model group.

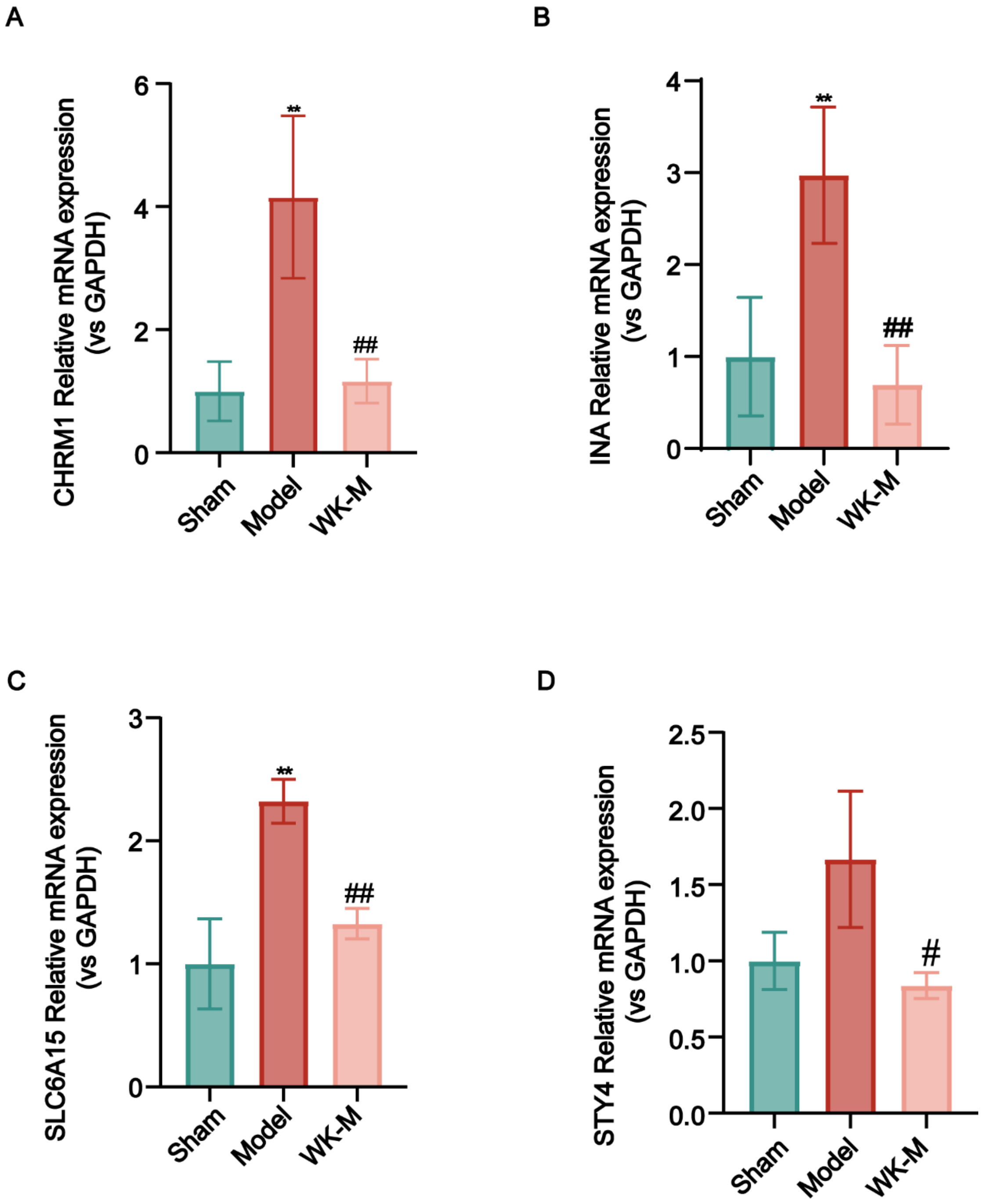

Figure S4. RT-PCR verification results of RNA-seq. (A-D) The mRNA expression levels of the CHRM1, INA, SLC6A15, and SYT4. Values are given as mean ± SD (n = 3, $^{*}P < 0.05$ and $^{**}P < 0.01$ vs. Sham group, $^{\#}P < 0.05$ and $^{\#\#}P < 0.01$ vs. Model group).

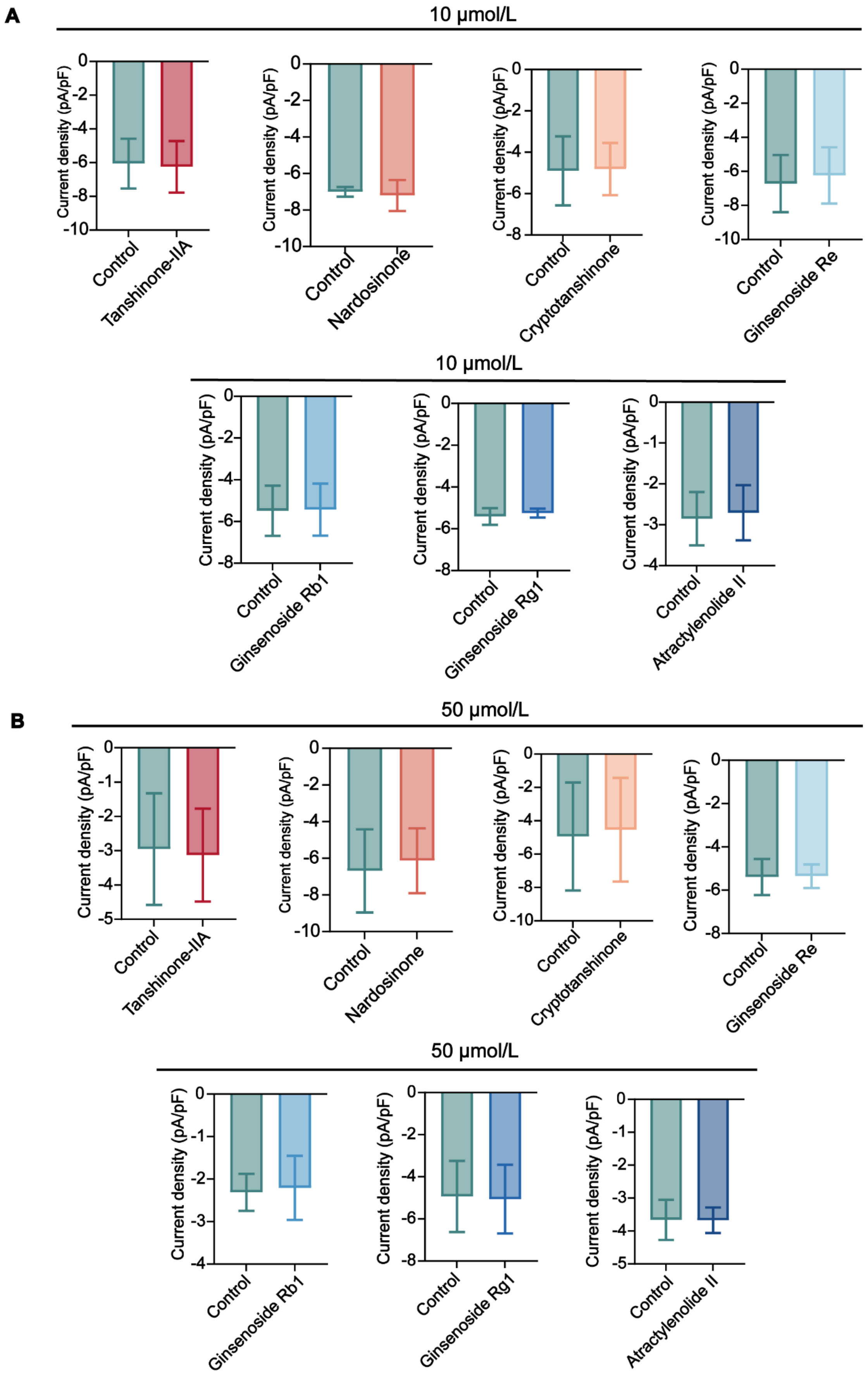

Figure S5. Statistical data for WK's active compounds. (A) Combined data of $I_{Ca\text{-}L}$ recorded

during exposure to Tanshinone-IIA (10 μmol/L), Nardosinone (10 μmol/L), Cryptotanshinone (10 μmol/L), Ginsenoside Re (10 μmol/L), Ginsenoside Rb1 (10 μmol/L), Ginsenoside Rg1 (10 μmol/L), Atractylenolide (10 μmol/L) and fushing under Control conditions. (B) Combined data of I_{Ca-L} recorded during exposure to Tanshinone-IIA (50 μmol/L), Nardosinone (50 μmol/L), Cryptotanshinone (50 μmol/L), Ginsenoside Re (50 μmol/L), Ginsenoside Rb1 (50 μmol/L), Ginsenoside Rg1 (50 μmol/L), Atractylenolide (50 μmol/L) and fushing under Control conditions. ($P>0.05$)

Supplementary data: **Table 1. Primer sequences of rat genes.**

Primer name		Primer sequence (5'−3')
INA	FORWARD	GGCTGAATCCACTTCCCAATCCC
INA	REVERSE	TCTTCCTCCTCCTCCTCCTCCTC
IFI44	FORWARD	AGTGCTCCGACTGCCTTCTCTC
IFI44	FORWARD	ACCAGGTCTCCATACGGCTTGTAG
CHRM1	FORWARD	GCCGCCTTCTACCTCCCTGTC
CHRM1	REVERSE	CTGCTGCTGCCACCACCTTTG
SLC6A15	FORWARD	TTGGGTTTGGGATTTGGTGGAGTC
SLC6A15	REVERSE	ACAGCGTCGAAGTGGCAGTTG
SYT4	FORWARD	ATCAGTCCACTACAAACACGCTCAC
SYT4	REVERSE	TCTTCTTGGCATGGTACAGGTTCAC
CHAT	FORWARD	AGCGAGCCTTGTTGACATGA
CHAT	REVERSE	GATTGCTTGGCTTGGTTGGG
SLC5A7	FORWARD	CCAAGACCAAGGAGGAAGCAGAC
SLC5A7	REVERSE	GCACCAAGCCCAAAGAAGGAAATG
CHRM4	FORWARD	CCGCACTACTAAGATGGCAGGTC
CHRM4	REVERSE	GGCAGGTAGAAGGCAGCAATGG
CACNA1B	FORWARD	GCCTTCGGGGTGTTGAACTA
CACNA1B	REVERSE	GTTCGTTTCCGCAATCTCCG
ACHE	FORWARD	AACAGAAGCCTCCGGATTGG
ACHE	REVERSE	ACCGACTTCCGAGCTCTCTA
CACNB1	FORWARD	AGGGCAAATACAGCAAGAGGAAAGG
CACNB1	REVERSE	GTTGGATGTGGTATCTGAGGAGGTG
ATP2A3	FORWARD	AGTCGGCAGCGGAGATGGTTC
ATP2A3	REVERSE	TTGGAGGAGATGAGGTAGCGGATG
GNG3	FORWARD	AGCACGCAAGATGGTGGAACAG
GNG3	REVERSE	GGGCATCACAGTAAGTCATCAGGTC
CHRM3	FORWARD	CGGTCGCTGTCACTTCTGGTTC
CHRM3	REVERSE	GTCGCTGCTGCTGTGGTCTTG
GAPDH	FORWARD	ACGGCAAGTTCAACGGCACAG
GAPDH	REVERSE	CGACATACTCAGCACCAGCATCAC

Method

Sample collection and preparation

RNA quantification and qualification

RNA integrity was assessed using the RNA Nano 6000 Assay Kit of the Bioanalyzer 2100 system (Agilent Technologies, CA, USA).

Library preparation for Transcriptome sequencing

Total RNA was used as input material for the RNA sample preparations. Briefly, mRNA was purified from total RNA using poly-T oligo-attached magnetic beads. Fragmentation was carried out using divalent cations under elevated temperature in First Strand Synthesis Reaction Buffer(5X). First strand cDNA was synthesized using random hexamer primer and M-MuLV Reverse Transcriptase (RNase H-). Second strand cDNA synthesis was subsequently performed using DNA Polymerase I and RNase H. Remaining overhangs were converted into blunt ends via exonuclease/polymerase activities. After adenylation of 3' ends of DNA fragments, Adaptor with hairpin loop structure were ligated to prepare for hybridization. In order to select cDNA fragments of preferentially 370~420 bp in length, the library fragments were purified with AMPure XP system (Beckman Coulter, Beverly, USA). Then PCR was performed with Phusion High-Fidelity DNA polymerase, Universal PCR primers and Index (X) Primer. At last, PCR products were purified (AMPure XP system) and library quality was assessed on the Agilent Bioanalyzer 2100 system.

Clustering and sequencing (Novogene Experimental Department)

The clustering of the index-coded samples was performed on a cBot Cluster Generation System using TruSeq PE Cluster Kit v3-cBot-HS (Illumia) according to the manufacturer's instructions. After cluster generation, the library preparations were sequenced on an Illumina Novaseq platform and 150 bp paired-end reads were generated.

Data Analysis

Quality control

Raw data (raw reads) of fastq format were firstly processed through fastp software. In this step, clean data (clean reads) were obtained by removing reads containing adapter, reads containing ploy-N and low-quality reads from raw data. At the same time, Q20, Q30 and GC content the clean data were calculated. All the downstream analyses were based on the clean data with high quality.

Reads mapping to the reference genome

Reference genome and gene model annotation files were downloaded from genome website directly. Index of the reference genome was built using Hisat2 v2.0.5 and paired-end clean reads were aligned to the reference genome using Hisat2 v2.0.5. We selected Hisat2 as the mapping tool for that Hisat2 can generate a database of splice junctions based on the gene model annotation file and thus a better mapping result than other non-splice mapping tools.

Novel transcripts prediction

The mapped reads of each sample were assembled by StringTie (v1.3.3b) in a reference-based approach. StringTie uses a novel network flow algorithm as well as an optional de novo assembly step to assemble and quantitate full length transcripts
representing multiple splice variants for each gene locus.

Quantification of gene expression level

featureCounts v1.5.0-p3 was used to count the reads numbers mapped to each gene. And then FPKM of each gene was calculated based on the length of the gene and reads count mapped to this gene.

FPKM, expected number of Fragments Per Kilobase of transcript sequence per Millions base pairs sequenced, considers the effect of sequencing depth and gene length for the reads count at the same time, and is currently the most commonly used method for estimating gene expression levels.

1. Sequencing error rate distribution

Sham group:

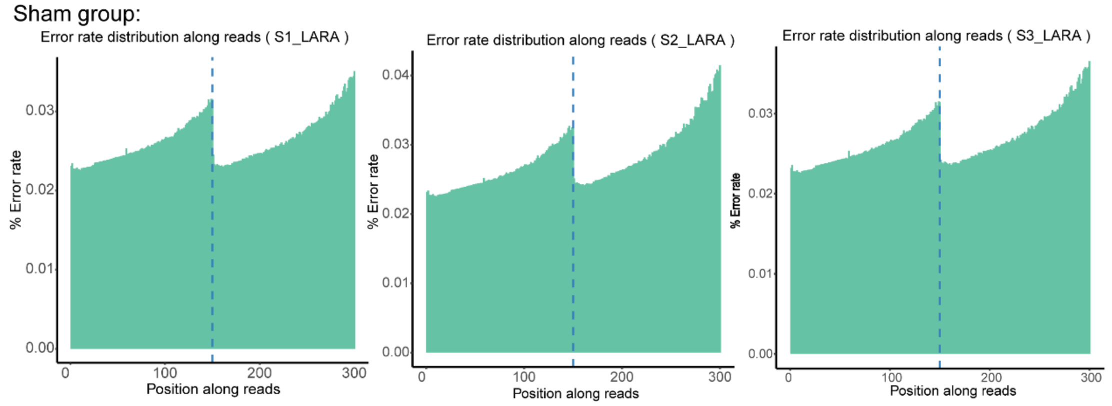

Model group:

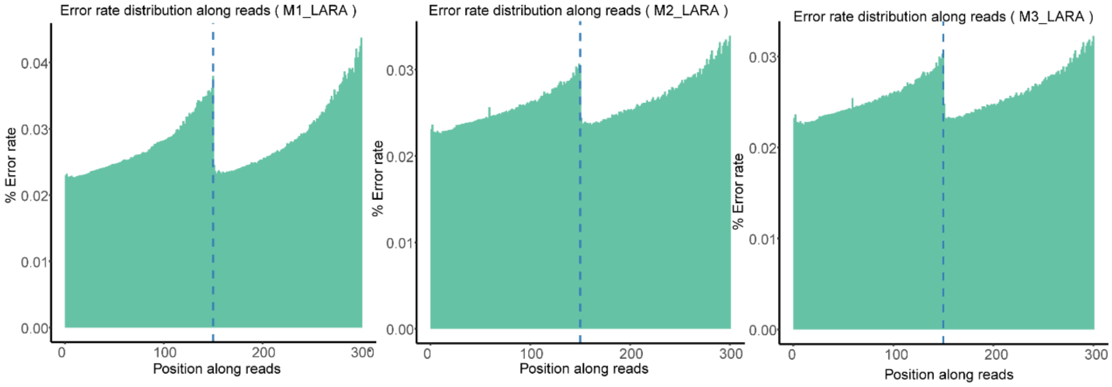

WK group:

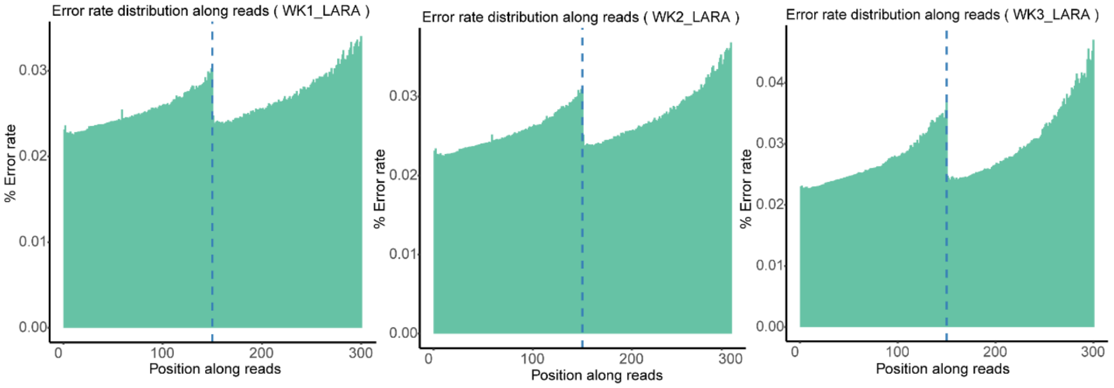

2. GC content distribution

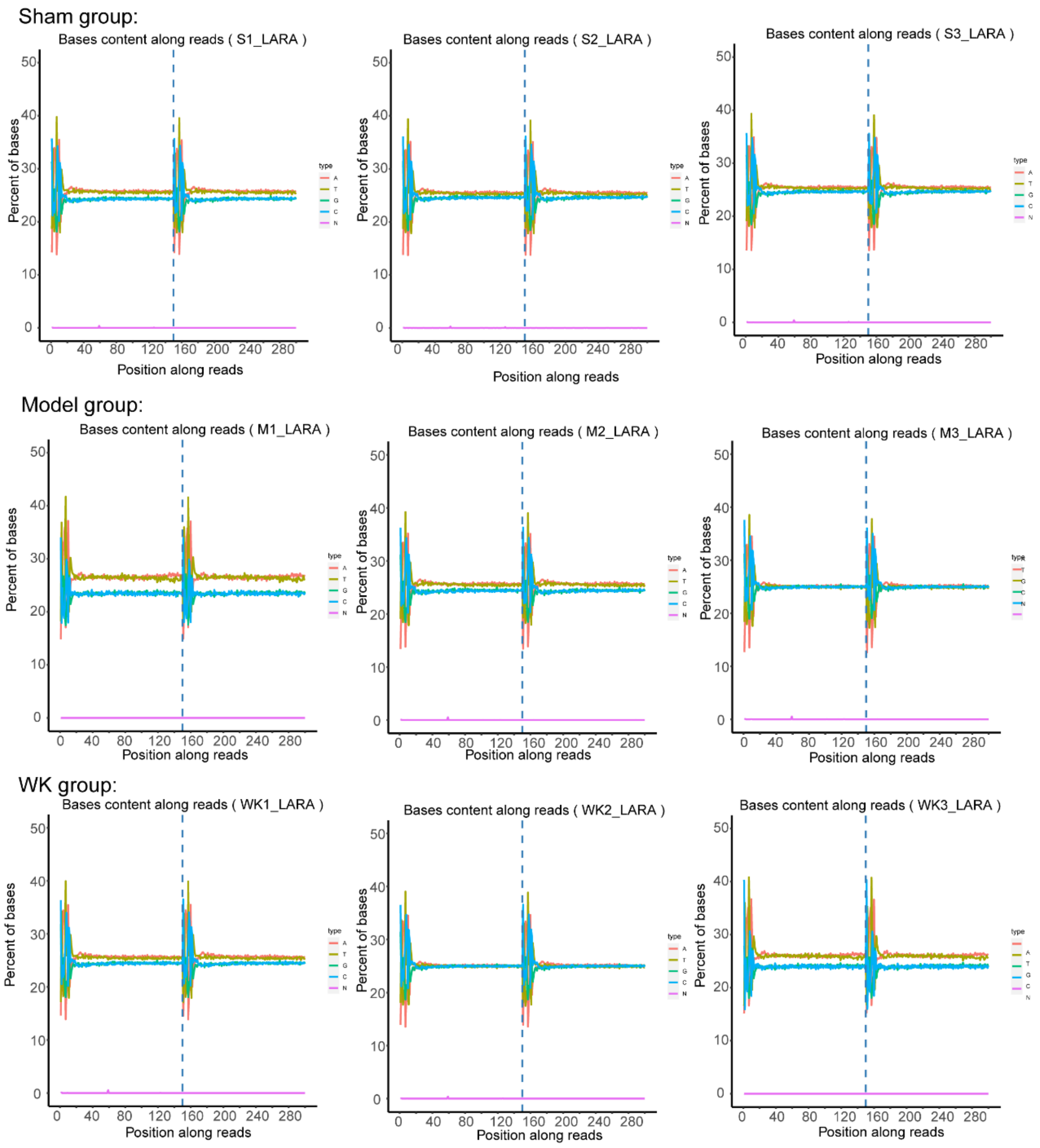

3. Sequencing data filtering

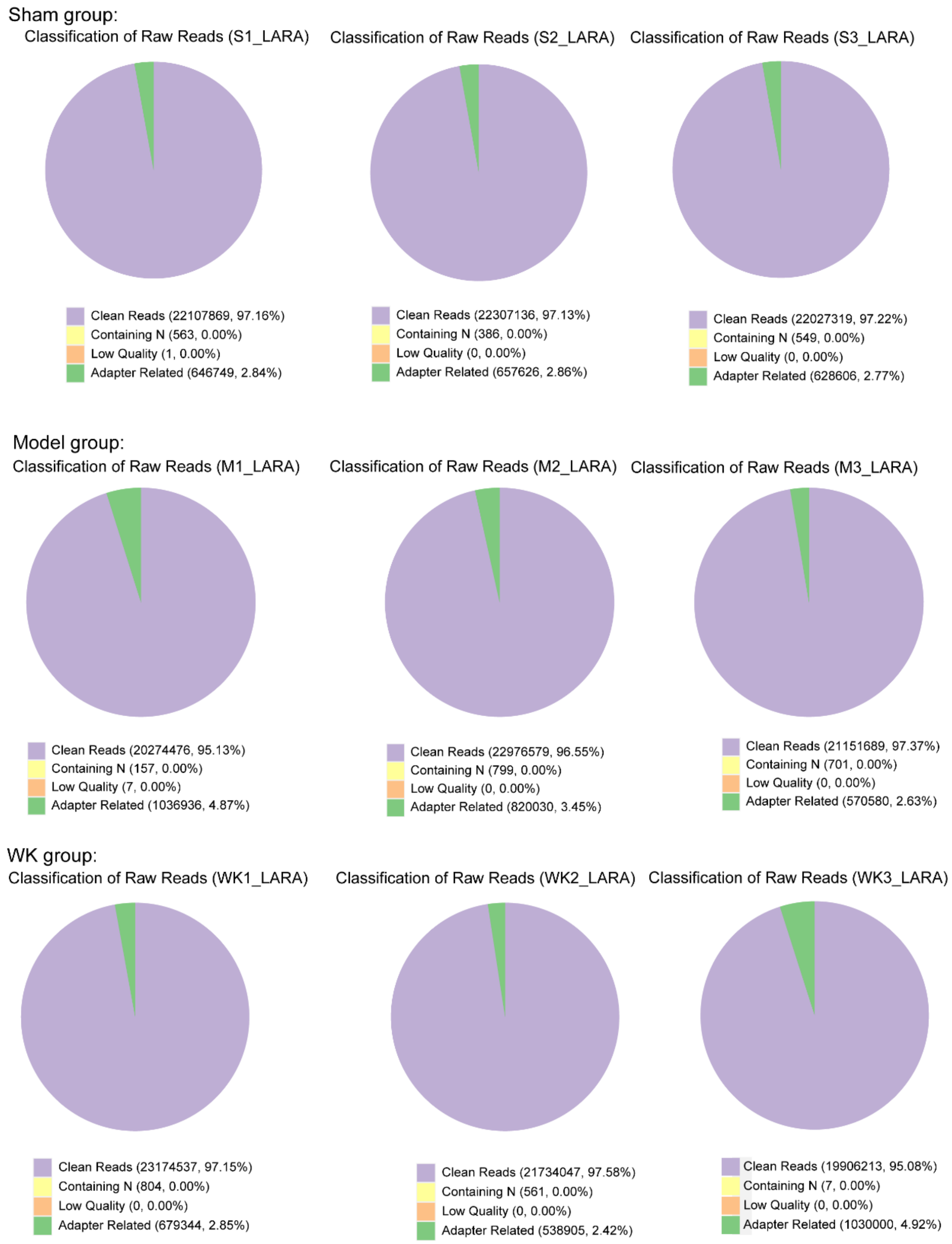

The Sequence Read Archive (SRA) is summarized in the : https://www.ncbi.nlm.nih.gov/sra/PRJNA1157589.
